# Unbiased Quantitative Models of Protein Translation Derived from Ribosome Profiling Data

**DOI:** 10.1371/journal.pcbi.1004336

**Published:** 2015-08-14

**Authors:** Alexey A. Gritsenko, Marc Hulsman, Marcel J. T. Reinders, Dick de Ridder

**Affiliations:** 1 The Delft Bioinformatics Lab, Department of Intelligent Systems, Delft University of Technology, Delft, The Netherlands; 2 Platform Green Synthetic Biology, Delft, The Netherlands; 3 Kluyver Centre for Genomics of Industrial Fermentation, Delft, The Netherlands; 4 Bioinformatics Group, Wageningen University, Wageningen, The Netherlands; The Pennsylvania State University, UNITED STATES

## Abstract

Translation of RNA to protein is a core process for any living organism. While for some steps of this process the effect on protein production is understood, a holistic understanding of translation still remains elusive. *In silico* modelling is a promising approach for elucidating the process of protein synthesis. Although a number of computational models of the process have been proposed, their application is limited by the assumptions they make. Ribosome profiling (RP), a relatively new sequencing-based technique capable of recording snapshots of the locations of actively translating ribosomes, is a promising source of information for deriving unbiased data-driven translation models. However, quantitative analysis of RP data is challenging due to high measurement variance and the inability to discriminate between the number of ribosomes measured on a gene and their speed of translation. We propose a solution in the form of a novel multi-scale interpretation of RP data that allows for deriving models with translation dynamics extracted from the snapshots. We demonstrate the usefulness of this approach by simultaneously determining for the first time per-codon translation elongation and per-gene translation initiation rates of *Saccharomyces cerevisiae* from RP data for two versions of the Totally Asymmetric Exclusion Process (TASEP) model of translation. We do this in an unbiased fashion, by fitting the models using only RP data with a novel optimization scheme based on Monte Carlo simulation to keep the problem tractable. The fitted models match the data significantly better than existing models and their predictions show better agreement with several independent protein abundance datasets than existing models. Results additionally indicate that the tRNA pool adaptation hypothesis is incomplete, with evidence suggesting that tRNA post-transcriptional modifications and codon context may play a role in determining codon elongation rates.

## Author Summary

Translation, the process of synthesizing proteins from mRNA templates, is an essential biological process in all living organisms. A better understanding of this process will have ramifications in various fields—from gene regulation, disease understanding and medicine to biotechnology and synthetic biology. Nonetheless, a holistic understanding of the processes remains elusive, making computational modelling a promising approach for studying it. However, accurate modelling of translation is challenging due to many assumptions made by such models and due to the sheer number of parameters that need to be specified. Here, we propose to fit models of translation onto ribosome profiling measurements, which record snapshots of the locations of actively translating ribosomes on mRNAs from millions of cells. We develop statistical and computational methods for fitting the Totally Asymmetric Exclusion Process (TASEP) models of translation on these measurements and verify them by deriving highly accurate translation models for the baker’s yeast *Saccharomyces cerevisiae*, which outperform existing models on independent datasets. We find that fitted elongation rate parameters from the derived models deviate significantly from the widely accepted tRNA pool adaptation hypothesis.

## Introduction

The process of protein synthesis is central to all living organisms. It has been actively researched for over five decades, and by now the individual steps of this process are known in great detail at the molecular and mechanistic levels [1]. Gene adaptation to the tRNA pool, mRNA secondary structure strength, codon order and local amino acid charge were independently implicated in shaping rates of protein production [2–4]. However, many disciplines would benefit from a holistic view of how these factors collectively influence translation. In particular, in biotechnology this knowledge would allow for tuning protein expression as desired with ramifications for cost-effective production of medicines and biofuels using microbes [5]. However, owing to the biological complexity of the process and the difficulty of measuring kinetic rates of the individual steps of protein synthesis, the development of computational models that would enable such applications lagged behind.

Only recently, the accumulated knowledge was integrated into several state-of-the-art models of increasing complexity. Zhang and Ignatova [6] proposed a “static” model for predicting the local speed of translation within a gene from codon-specific elongation rates derived from tRNA concentrations; their approach was extended by Reuveni *et al*. [7], who suggested using a “dynamic” model in which ribosomes initiate translation at the first codon and block each other while moving towards the end of the mRNA transcript. Siwiak and Zielenkiewicz [8] and Shah *et al*. [9] independently proposed static and dynamic full-cell models that additionally integrated the intracellular concentrations of ribosomes, mRNA and tRNA molecules, and their diffusion inside the cell in a single model. While predictions made by these models are usually in accordance with the current understanding of translation, most of their core assumptions (e.g. codon translation rates) have not been subjected to comparison against measured data.

Ribosome profiling (RP) [10, 11], a relatively new technique based on high-throughput sequencing of ribosome-protected RNA fragments (footprints), is nowadays often employed for studying translation [12–15]. It provides noisy snapshots of the locations of actively translating ribosomes attached to mRNA transcripts, thereby convolving the number of ribosomes and their speed of translation (a few stalled ribosomes can generate similar sets of footprints as many ribosomes involved in rapid translation). While in principle these data allow for simultaneously reasoning about ribosome counts and their local speed, such analysis is hampered by the limited understanding of the error model and biases of RP data [16]. To date RP measurements have been analyzed either at the level of full genes [8, 9] or at single codon resolution [4, 17]. While only the latter allows for analyzing the dynamics of translation, it is not clear whether codon-resolution measurements are sufficiently reliable for such quantitative analysis (see S1 Text). To overcome the measurement reliability issue several studies [18–20] performed “meta-codon” analysis by pooling observations from different occurrences of a particular codon together to produce an estimate of the codon elongation time. It is unclear, however, to what extent such estimates are affected by ribosomal interference.

**Fig 1:**
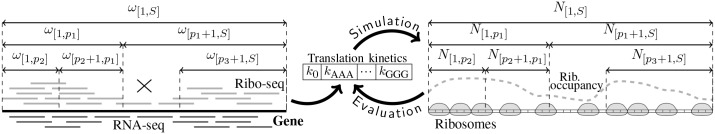
Schematic overview of the proposed approach for inferring translation kinetics from RP data. To obtain a segment tree representation of the RP data (left) mapped ribo-seq (light grey) and RNA-seq (dark grey) reads are assigned to nested segments of decreasing lengths (starting from segments [1, *S*] equivalent to the full-length CDSes) while there is sufficient data. Ribosome densities *ω* for each segment are computed for the available replicates and are used to parameterize the log-normal distributions describing measurement error of these segments. To determine per-gene translation initiation rates *k*_0_ and per-codon elongation rates *k*_AAA_, …, *k*_GGG_ many candidate sets of translation rates are tested. For every candidate set the TASEP model of translation is simulated with the proposed rates for all genes in the *model simulation step* (right). Ribosome occupancy, i.e. the relative amount of time ribosomes spend at a particular location on the mRNA, obtained from the simulation (dashed grey) is then aggregated per segment to compute the average occupancies *N*, which are compared the log-normal distributions of the corresponding segments from the segment tree representation in the *model evaluation step*. Evaluation results are used by a genetic algorithm to propose new candidate sets of rates and repeat the simulation-evaluation cycle until the search for translation rates converges. To simplify notation, the gene index *g* is dropped for all gene-specific variables in the figure.

We propose a set of methods for deriving full translation kinetics of an organism from RP data (see Fig 1). Our approach is conceptually similar to Ciandrini *et al*. [21], who inferred translation initiation rates of yeast genes from polysome profiling data, except that we use RP for deriving these rates and additionally determine the translation elongation rates. The method is based on a novel “segment tree” multi-scale interpretation of the RP data that captures ribosome translation dynamics along mRNAs without sacrificing reliability due to measurement noise. We use this interpretation to simultaneously extract, for the first time, per-gene translation initiation rates and per-codon translation elongation rates for the bakers yeast *Saccharomyces cerevisiae* by fitting two version of the TASEP (Totally Asymmetric Exclusion Process), a simple dynamic model of translation [22], on the segment tree estimates. To make fitting tractable, we devised a highly efficient initiation rate approximation scheme and combined it with a novel Monte Carlo simulation strategy inside an evolutionary optimization algorithm.

Fitted TASEP models match the RP data significantly better than the state-of-the-art models, and their predicted protein production rates are confirmed by several independent protein abundance (PA) datasets. In particular our models show significantly better agreement with PA than existing models when the measurements are corrected for mRNA levels, i.e. when only the effect of translation on protein levels is considered. Interestingly, the fitted codon elongation rates deviate significantly from the tRNA pool adaptation hypothesis.

## Materials and Methods

### Ribosome profiling data

RP data for yeast *Saccharomyces cerevisiae* strain S288C [23] containing ribosome footprint reads (ribo-seq) and fragmented mRNA reads (RNA-seq) measured in duplicate were obtained from the NCBI Short Read Archive (accession SRP028552). Reads were trimmed and mapped to the latest *S. cerevisiae* strain S288C reference genome taken from the Saccharomyces Genome Database (SGD, Cherry *et al*. [24]) in two stages, and assigned to gene coding sequences (CDSes) obtained from SGD. Aligned ribosome footprint and mRNA reads were assigned to single positions within the CDSes based on respectively their inferred A-sites or the centre position of the read (see S1 Text for details).

### Measurement resolution

To obtain a high-resolution map of mRNA and ribosome density without sacrificing measurement accuracy, for each gene we construct a segment tree of density measurements from nested parts of the CDSes (Fig 1, left). By pooling reads from all segment positions, average densities per segment can be calculated more reliably than would be possible at single codon resolution (see also S1 Text), while recording these densities for nested segments of decreasing lengths allows for indirectly capturing the change in density along a transcript.

Starting from an initial segment [*l*, *r*] equivalent to the complete CDS we count the number of ribo-seq reads *R*_[*l*,*r*]_ and RNA-seq reads *M*_[*l*,*r*]_ assigned to this segment. These counts are normalized by the total number of ribo- and RNA-seq reads aligned to all CDSes (*N*_*R*_ and *N*_*M*_ respectively) and the segment length *L*_[*l*,*r*]_ = *r* − *l* + 1 to obtain ribosome and mRNA densities $d_{\left[l,r\right]}^{\text{Ribo}}=\frac{R_{\left[l,r\right]}}{L_{\left[l,r\right]}N_{R}}$ and $d_{\left[l,r\right]}^{\text{mRNA}}=\frac{M_{\left[l,r\right]}}{L_{\left[l,r\right]}N_{M}}$ for the current segment. To obtain the sought *per transcript* ribosome density (later referred to as density ratio) the ratio of the two measurements $\omega_{\left[l,r\right]}=\frac{d_{\left[l,r\right]}^{\text{Ribo}}}{d_{\left[l,r\right]}^{\text{mRNA}}}$ is calculated. The average segment ribosome density given by this ratio is normalized for transcript abundance and allows for directly comparing segments from different genes to each other. A cut point *p* is then chosen and the process is repeated recursively for segments [*l*, *p*] and [*p* + 1, *r*] (see Fig 1, left). The aim behind calculating $d_{\left[l,r\right]}^{\text{mRNA}}$ for each segment independently instead of estimating a single gene-specific value is to remove any local sequencing bias (presumed to be identical between RNA- and ribo-seq since very similar protocols are used for library preparation [23]) from the ratio estimates. Density measurements are computed for each replicate individually, but the same segment cut points are used in order to merge replicates later. Cut points are chosen such that the combined number of RNA- and ribo-seq reads across replicates is divided equally between the left and the right segments (see S1 Text for details).

The recursive tree construction continues while there are sufficient reads for making reliable density estimates (at least 128 reads in the two replicates summed together for RNA-seq and ribo-seq, separately; see S1 Text for details on choosing these thresholds) and segment length is large enough, *L*_[*l*,*r*]_ ≥ 20 codons. The segment length cutoff aims at keeping the segments long enough to average out any measurement error due to incorrect read assignment or sequence bias. Prior to interpreting the measurements, we additionally remove a systematic density-dependent bias present in the density and ratio measurements using the available replicate information (see S1 Text).

This procedure was used to construct segment trees for 4, 892 genes with a total of 60, 466 nested density estimates left after removing genes classified as dubious or located on the mitochondrial chromosome.

### Statistical treatment of the measurements

In order to accurately capture variance of RP data, we assume that the measured segment densities follow a log-normal distribution around the density values. A similar assumption is often made for transcriptome measurements and is justified by the observation that inter-replicate errors (i.r.e.), i.e. the ratios of replicated mRNA and ribosome density measurements, follow a log-normal distribution (S1 Fig and Ingolia *et al*. [10]). It then holds that density ratios *ω*_[*l*_*j*_,*r*_*j*_]_ (*j* ∈ *J* ^*g*^, where *J* ^*g*^ is the set of all segments of gene *g*) from different replicates also follow a log-normal distribution ln𝓝(*μ*_*j*_, *σ*_*j*_) as ratios of log-normally distributed random variables—the mRNA and ribosome segment densities. Here *μ*_*j*_ and *σ*_*j*_ are used as shorthands for *μ*_[*l*_*j*_,*r*_*j*_]_ and *σ*_[*l*_*j*_,*r*_*j*_]_ respectively.

To determine the parameters of this distribution we estimate *μ*_*j*_ for the *j*-th segment from the available replicated measurements as the log of their geometrical mean. Ideally, a separate shape parameter *σ*_*j*_ should also be estimated per segment, but, given the number of replicates, doing so would not yield reliable estimates. Instead it was chosen to group segments from all genes based on their length, and estimate shape parameters $\sigma_{k}^{\text{group}}$ for group *k* from the i.r.e. of measurements from that group (see S1 Text and S2 Fig).

The proposed measurement distribution $\text{ln}𝓝(\mu_{j},\sigma_{k_{j}}^{\text{group}})$, where *k*_*j*_ denotes the length group of the *j*-th segment, is used throughout this paper as an error model for fitting TASEP models of translation on RP data and for comparing different models with the data.

### Data interpretation and model evaluation

Computational models of translation typically provide the ability to extract steady-state codon occupancy probabilities obtained from model simulations, i.e. estimates of the chance that a particular position of an mRNA is occupied by an actively translating ribosome. Similar to the ribosome profiling measurements these occupancy profiles are determined by the local speed of translation and the number of ribosomes translating an mRNA. This allows for evaluating how well a given model matches the RP data by comparing the average segment occupancies and the segment tree ratio estimates (see Fig 1, right).

Quantitative measurements obtained via high-throughput sequencing such as the mRNA and ribosome densities (and hence their ratios) are measured in arbitrary units. Without explicit assumptions on the physiological characteristics of the analyzed organism, such as the full size of its transcriptome [8] or the number of ribosomes per cell [9], and on the efficiency of individual experimental steps, it is impossible to estimate sequencing depth of the RP measurements (i.e. the average number of reads per ribosome or the average number of reads per kilobase of transcript) and therefore impossible to express the measured values in physiologically meaningful units (e.g. number of ribosomes per transcript). Additionally, this unit mismatch complicates the comparison of modeled ribosome occupancies to the measured densities. To derive a model evaluation criterion, we first assume that an unknown scaling factor *C* that transforms model output into measurement data units is given, and propose a method for calculating it later.

Let $n_{i}^{g}$ be the model-predicted ribosome occupancy at position *i* of gene *g* and $T^{g}=\left\{\left(\mu_{j}^{g},\sigma_{j}^{g}\right)|j\in J^{g}\right\}$ be the set of ratio distribution parameters for segments $\left[l_{j}^{g},r_{j}^{g}\right]$. Here the upper index *g* denotes the gene, and for a more succinct notation we use the lower index *j* in place of $\left[l_{j}^{g},r_{j}^{g}\right]$. For segment *j* on gene *g* the probability of the predicted occupancies given the segment ratio estimates can be expressed as

$$p\;(C,N_{j}^{g}|\mu_{j}^{g},\sigma_{j}^{g})\propto f_{C}\;\left(N_{j}^{g};\mu_{j}^{g},\sigma_{j}^{g}\right),$$ (1)

where $N_{j}^{g}\equiv \sum_{i=l_{j}^{g}}^{r_{j}^{g}}\;n_{i}^{g}/\left(r_{j}^{g}-l_{j}^{g}+1\right)\;$ is the predicted average occupancy on segment *j* of gene *g*, and $f_{C}(x;\mu ,\sigma )=\frac{1}{x\sigma \sqrt{2\pi }}e^{-\frac{{\left(\text{ln}\;x+\text{ln}\;C-\mu \right)}^{2}}{2\sigma^{2}}}$ is the log-normal probability density function describing the density ratio measurement error scaled by factor $\frac{1}{C}$. This formulation is used for comparing the predicted occupancies to the estimated values in a probabilistic fashion. Assuming independence between ratio estimates of the same gene and between genes, the probability of observing all estimates, denoted by *n*, can be expressed as

$$p\;(C,n|T)\propto \prod_{g}\prod_{j\in J^{g}}f_{C}\;\left(N_{j}^{g};\mu_{j}^{g},\sigma_{j}^{g}\right),$$ (2)

In practice these calculations are more easily performed in log space and the constant factors are dropped:

$$\psi (C,n|T)=\sum_{g}\sum_{j\in J^{g}}\ln f_{C}\left(N_{j}^{g};\mu_{j}^{g},\sigma_{j}^{g}\right)∼\sum_{g}\sum_{j\in J^{g}}[-\frac{1}{2{\left(\sigma_{j}^{g}\right)}^{2}}{\left(\ln N_{j}^{g}-\mu_{j}^{g}+\ln C\right)}^{2}-\ln N_{j}^{g}]$$ (3)

We use *ψ*(*C*, *n*∣*T*) as the objective function for quantifying how well model-predicted ribosome occupancies match measured data.

To choose the scaling factor *C*, we note that it is the only free parameter of *ψ*(*C*, *n*∣*T*) if model output *n* and segment tree estimates *T* are given. In that case, the value of *C* maximizing *ψ* can be determined analytically:

$$\ln C=(\sum_{g,j\in J^{g}}\frac{1}{{\left(\sigma_{j}^{g}\right)}^{2}}\;(\mu_{j}^{g}-\ln N_{j}^{g}))/(\sum_{g,j\in J^{g}}\frac{1}{{\left(\sigma_{j}^{g}\right)}^{2}})$$ (4)

Throughout this paper, different models are evaluated at a scaling factor *C* maximizing their fit to the data (i.e. maximizing *ψ*). While the unknown true scaling factor is determined by the physiological properties of the cell, the efficiency of the experimental protocols and characteristics of the high-throughput sequencing measurements (see section “Initiation rate approximation” and S1 Text), evaluating models at the best possible scale allows for a more fair evaluation as it does not penalize models in cases when the model and the true scales mismatch.

### The TASEP model of translation

**Fig 2:**
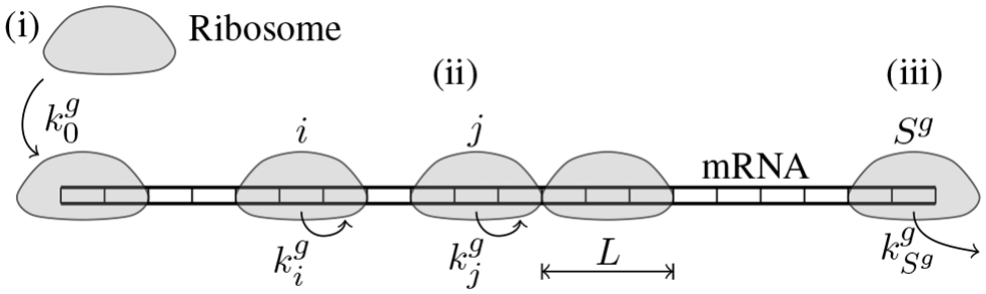
In TASEP mRNAs are modeled as one-dimensional lattices of *S* ^*g*^ sites (codons) and ribosomes—as particles occupying *L* sites (*L* = 3 in the figure). During translation (i) ribosomes attach to the first codon with rate $k_{0}^{g}$ if the beginning of the mRNA is not occupied by other ribosomes (initiation); (ii) ribosomes move from position *i* to *i* + 1 with a site-specific rate $k_{i}^{g}$ if not blocked by another ribosome (elongation); and, finally, (iii) after reaching the last codon, they detach with rate $k_{S^{g}}^{g}$ (termination).

TASEP (Totally Asymmetric Exclusion Process) models mRNAs *g* as one-dimensional lattices of length *S* ^*g*^ and ribosomes as abstract “particles” occupying *L* sites corresponding to codons (Fig 2). These particles hop on (translation initiation) and off (translation termination) the lattice at the first and last sites with rates $k_{0}^{g}$ and $k_{S^{g}}^{g}$ respectively. They only move towards the end of the lattice (hence the totally asymmetric) by hopping one site at a time with site-specific elongation rate $k_{i}^{g}$. Ribosomes interact with each other by “excluding” a volume of *L* sites that they cover on the lattice, meaning that a ribosome cannot continue to the next codon if it is already covered by another ribosome. The exact location of the active site among the *L* covered codons does not change the rules governing ribosome motion [22], but the choice of *L* may influence simulation dynamics in cases of high ribosome queueing. Typically, values 9 ≤ *L* ≤ 11 are used [8, 9, 16, 21]; *L* = 10 was chosen for our simulations as it best matches the RP footprint size distribution [10].

TASEP captures the high-level physical interaction between ribosomes and transcripts by describing the ribosomes as travelling on the mRNAs. While in practice a number of varying translation scenarios are possible (e.g. RER-bound translation with ribosomes glued to the endoplasmic reticulum and moving very slowly while the mRNA is instead pulled though the ribosomes [25]), the rich set of behaviors attainable by TASEP makes it a suitable framework for modelling translation. It requires specification of a large number of parameters, namely the gene- and site-specific elongation rates $k_{i}^{g}$ (with the stop codon elongation rate functioning as the termination rate) and the gene-specific initiation rates $k_{0}^{g}$. To reduce the number of parameters we assume that the site-specific elongation rates are codon-specific and do not differ between genes. This commonly made assumption [7, 16, 21, 26] is necessary for determining model parameters from RP data as it makes the model fitting problem tractable. Depending on the experiment, either elongation rates consistent with the tRNA pool adaptation hypothesis were fixed to allow fitting the initiation rates only, or all model parameters were fit on the available data.

### Monte Carlo simulations

Evaluation and fitting of the TASEP model requires an efficient way of obtaining steady-state ribosome occupancies. TASEP models allow limited analytical tractability and, to our knowledge, no analytical results for the steady-state codon occupancy probabilities are available for the general case. Additionally, existing TASEP mean-field approaches poorly approximate codon occupancies [27], a quantity of particular importance to this study, leaving stochastic simulations as the only suitable approach.

TASEP steady-state codon occupancies were obtained by simulating the model using a Monte Carlo algorithm, i.e. by randomly selecting an event (translation initiation, elongation or termination) in every simulation step and, if no other ribosomes interfere with the event, executing it with a probability proportional to its rate. To speed up simulation we developed a continuous time simulation method similar to the Gillespie algorithm [28], but based on the use of the Erlang distribution to only sample times between *state-changing events*, i.e. events that change the configuration of ribosomes attached to an mRNA.

Formally, the times between consecutive initiation or elongation events at position *i* are assumed to be exponentially distributed with rates $k_{0}^{g}$ and $k_{i}^{g}$ respectively (i.e. the corresponding model rate parameters, Fig 2). Let *o*_*i*_, *i* = 1, …, *S* ^*g*^ be the current state of the simulated molecule:

$$o_{i}=\{\begin{array}{cc} 1, & \text{codon}\;i\;\text{is}\;\text{occupied}\;\text{by}\;\text{a}\;\text{ribosome}\;(\text{is}\;\text{at}\;\text{its}\;\text{A-site})\\ 0, & \text{otherwise} \end{array}.$$ (5)

Then the time between *any* two consecutive events is also exponentially distributed with rate $k=k_{0}^{g}+\sum_{i=1}^{S^{g}}o_{i}k_{i}^{g}$ as the minimum of independent exponentially distributed random variables. Once an event occurred, the probability that it was event *j* is given by $p_{j}=\;o_{j}k_{j}^{g}/k\;$ (it is assumed that ribosomes are always available to initiation translation, i.e. *o*_0_ = 1). Some of the events cannot be executed due to ribosomes blocking each other and do not lead to a state change. If *k*_+_ is the sum of rates of events leading to a state change, then the number of events between consecutive state changes, denoted as *e*, follows a geometric distribution with parameter *p*_+_ = *k*_+_/*k* and the time Δ*t* between state changing events follows the Erlang distribution with shape *e* and rate *k* as the sum of iid exponential random variables. The simulation proceeds by repeated random sampling of the number of events, the time between events and the event type *s* from the appropriate probability distributions; and updating ribosome locations in accordance to the sampled event:

$$\begin{array}{ccc} s∼\text{Categorial}\;(p_{0},p_{1},\ldots ,p_{S^{g}}), & e∼\text{Geometric}\;(p_{+}), & \Delta t∼\text{Erlang}\;(e,k). \end{array}$$ (6)

Simulating only state-changing events allows the simulation to progress faster, especially in cases of high ribosome queueing. The total time $T_{i}^{g}$ spent by ribosomes at position *i* and the total simulation time *T* ^*g*^ are recorded to estimate the per-transcript ribosome occupancy at this position as $n_{i}^{g}=\;T_{i}^{g}/T^{g}\;$, which is then used for comparing the model to RP data. Similarly the total number of translation terminations *F* ^*g*^ is used to estimate the protein production rate *J* ^*g*^ = *F* ^*g*^/*T* ^*g*^.

To reach steady-state distribution of ribosomes on mRNA irrespective of the CDS length, each mRNA was simulated until 1000 translation termination events occurred. After that the model was further simulated for up to 10^7^ additional steps or until the average ribosome occupancy in the segments of interest was estimated with high precision (absolute error *ϵ* < 10^−3^). The latter stopping criterion is based on the observation that average ribosome occupancy over a fixed segment of the mRNA can be reliably estimated before per-position occupancies can. Segment densities were first estimated after 5 × 10^5^ simulation steps and then every 10^6^ steps. Simulation was stopped if the absolute error between consecutive estimates was smaller than *ϵ*.

### Initiation rate approximation

In addition to the elongation rates, large TASEP models require specification of hundreds gene translation initiation rates prior to simulation. Direct measurements of the initiation rates rates are unavailable and instead their values are often inferred from other sources such as ribosome profiling [8, 9] or polysome size measurements [21] data. Initiation rates estimated in such a way depend on the rates of translation elongation used in the analysis, and hence need to be optimized together with the elongation rates of the TASEP model. This leads to an explosion of the number of parameters that need to be determined, stressing the need for highly efficient initiation rate approximation strategies if the initiation and elongation rates are to be determined from the RP data simultaneously.

The problem of determining initiation rates was previously tackled by approximations neglecting ribosome queueing [8, 9], and by near-exhaustive computational search [21]. We propose a method that is a compromise between the two approaches—it allows approximating gene initiation rates for the TASEP model from RP data at a fraction of the computational cost of an exhaustive search. Briefly, we add an additional parameter $\tilde{C}$, the “proposed” scaling factor, to the list of model parameters that need to be estimated. This parameter is identical to the scaling factor *C* from Eq (4), but is used within the model to obtain biologically meaningful initiation rates. We calculated the value of $\tilde{C}$ from the number actively translating ribosomes [29] and the number of mRNA molecules [30] per cell using a procedure proposed by Siwiak and Zielenkiewicz [8]. Given some estimate of the elongation rates and $\tilde{C}$ we then find optimal initiation rates using a novel numerical approximation of ribosome density for TASEP models that is based on the observations of Cinandrini *et al*. [21]. This approach allows us to decouple initiation rates from elongation rates and greatly reduces the number of model parameters that need to be fitted explicitly (next section). We used this method to efficiently (re-)approximate initiation rates of genes for each new set of elongation rates $k_{i}^{g}$. A full description of the approach is available in the S1 Text.

### Model fitting

When fitting the TASEP models, translation rates that maximize *ψ*(*C*, *n*∣*T*) are sought. Lacking a closed-form solution, we employed the Covariance Matrix Adaptation Evolutionary Strategy (CMA-ES [31]) to find these rates.

We considered two different TASEP models: TASEP^init^ and TASEP^elong^. In TASEP^init^ the elongation rates are fixed at values consistent with the tRNA pool adaptation hypothesis and initiation rates are approximated as described earlier. In the TASEP^elong^ model none of the parameters are fixed: also the codon-specific elongation rates are optimized with the CMA.

Since TASEP simulation output is invariant to scaling of translation rates, many equally good solutions exist. To constrain the search the elongation rate of codon GAA was fixed at its initial tRNA pool adaptation hypothesis value. The codon was chosen as it is present in many genes and segments (S5 Fig). Further details regarding the use of CMA can be found in the S1 Text.

Despite the efficient Monte Carlo simulation and translation rate search strategies, model fitting remains a very CPU-intensive task. To speed up computations in practice, the models were fitted using hundreds of CPUs in parallel as individual genes can be simulated independently.

Because TASEP simulations of different genes are independent of each other, it may be unclear how to interpret the fitted elongation and initiation rates, as they must depend on such global biophysical quantities as the number of tRNAs or ribosomes within the cell. Nevertheless, the final simulation results are compared to whole-genome RP measurements. We can therefore expect that if our TASEP simulations agree well with RP data, the fitted translation rates used within the simulations account for the necessary biophysical parameters. Thus they should be regarded as the *effective* initiation and elongation rates that account for the relevant biophysical characteristics of the cell and growth conditions. We note that translation rates determined in such a way are condition-specific, and would likely change if fitted on a dataset obtained under different growth conditions.

### Comparison to other models

To obtain a baseline for evaluating the performance of fitted TASEP models we also evaluated several existing state-of-the-art static and dynamic models of translation and compared them to each other based on their agreement with the RP data as given by Eq (1). SMoPT [9] and Zhang’s model [6] were chosen for evaluation on the segment tree data as other state-of-the-art models, namely the Ribosome Flow Model [7] and the model from Siwiak and Zielenkiewicz [8], do not provide ribosome occupancy profiles compatible with the segment tree interpretation. The latter model was however compared to the fitted TASEP models based on several independent PA datasets.

When comparing models’ predictions using independent protein abundance datasets, the “initiation frequency” *P*, “total amount of protein molecules produced from transcripts of particular type” *B* and the “total time of translation of one protein molecule from a given transcript” *T* from Siwiak and Zielenkiewicz [8] were respectively treated as translation initiation rate, the product of *J* and mRNA levels, and the inverse of *J*; the average gene total elongation time from SMoPT [9] was treated as the inverse of *J*; 𝓟 from Ciandrini *et al*. [21] was treated as *J*.

### Experimental setup

Since the sets of genes included in SMoPT and the segment trees differ, to facilitate comparison, all models were evaluated on a set of 3, 617 genes (49, 894 segments) that were in common between all models after removing very long genes to speed up TASEP simulations (31 genes longer than 2, 000 codons). This set of genes was used to fit TASEP models inside a 5-fold stratified cross-validation (CV) loop over genes, in which the CV folds were chosen to balance the number of genes and segments between folds. In every step of the CV 1 fold was used for fitting (training set) and 4 folds were used for model evaluation (test set). Smaller training sets were used to reduce model fitting time. To evaluate predictions of the proposed TASEP models we also fitted them on all segment tree estimates. And to further reduce fitting time on this large dataset, codon elongation rates of the TASEP^elong^ model were set to the geometric mean of elongation rates from CV folds, and only the initiation rates were estimated from the data.

To simplify comparison of different models, we computed CV objectives for all evaluated models, including the models that did not require any parameter fitting (i.e. SMoPT and Zhang’s model). While the static Zhang model does not explicitly model the translation initiation step, SMoPT and TASEP models require initiation rates to be defined for every gene in the test sets in order to calculate the CV objective. We used the original initiation rates inferred from the RP data [9, 10] for SMoPT, and approximated TASEP initiation rates using the test set segment tree measurements.

### The tRNA pool adaptation hypothesis

Some of the experiments required the translation elongation rates to be defined. For those experiments we used translation elongation rates *k*_AAA_, …, *k*_GGG_ consistent with the tRNA pool adaptation hypothesis, which could be seen as a statement that codons recognized by more abundant tRNAs are translated faster. The exact values for the elongation rates were defined based on the tRNA Adaptation Index (tAI [32]), which quantifies the decoding efficiency of a codon by simultaneously considering abundances of all tRNA species recognizing it and the strength of wobble base pairing between the codon and the anticodons of the isoacceptor tRNAs. The elongation rates *k*_AAA_, …, *k*_GGG_ were calculated as the inverse of the codon translation times taken from the Ribosome Flow Model [33]; and translation termination rates (i.e. *k*_TAG_, *k*_TAA_, *k*_TGA_) were set to 1.

### Comparison to tAI and CAI

The tAI and CAI (Codon Adaptation Index [34]) are the most commonly used codon indices. They quantify respectively the extent to which a particular sequence consists of codons recognized by abundant tRNAs, and the extent to which a particular sequence consists of codons present in highly expressed (e.g. ribosomal and glycolytic) genes. These indices are often used as a proxy for translational efficiency of a gene and are employed to optimize its sequence for expression in a different host organism. Having determined elongation rates for the TASEP^elong^ model, we sought to understand whether these rates suggest a different optimization scheme than the one given by tAI or CAI.

For each codon the tAI (CAI) assigns a number—the absolute adaptiveness of that codon to the tRNA pool (codons used in highly expressed genes). To facilitate comparison between the different indices, following the definition of the CAI, we define the relative adaptiveness of a codon as its absolute adaptiveness normalized by the maximum adaptiveness among synonymous codons. We then use the relative adaptiveness for CAI, tAI and an index based on the TASEP^elong^ elongation rates (described below), when comparing optimization schemes.

We note that from the definitions of tAI [32] and elongation rates consistent with the tRNA pool hypothesis (previous section and [7]) it follows that the tAI absolute codon adaptiveness and the elongation rates are proportional to each other, and use this observation to define a codon index based on the fitted TASEP^elong^ elongation rates. We define the relative adaptiveness of a codon according to TASEP^elong^ as its elongation rate normalized as described above.

### Other datasets

Protein abundance measurements were taken from Newman *et al*. [35] (YEPD and SD media) and Ghaemmaghami *et al*. [36]. 5′- and 3′ UTR lengths were determined based on Nagalakshmi *et al*. [37] and Yassour *et al*. [38] as the mean length obtained from the two studies.

## Results

### Segment trees reliably capture density changes along transcripts

**Fig 3:**
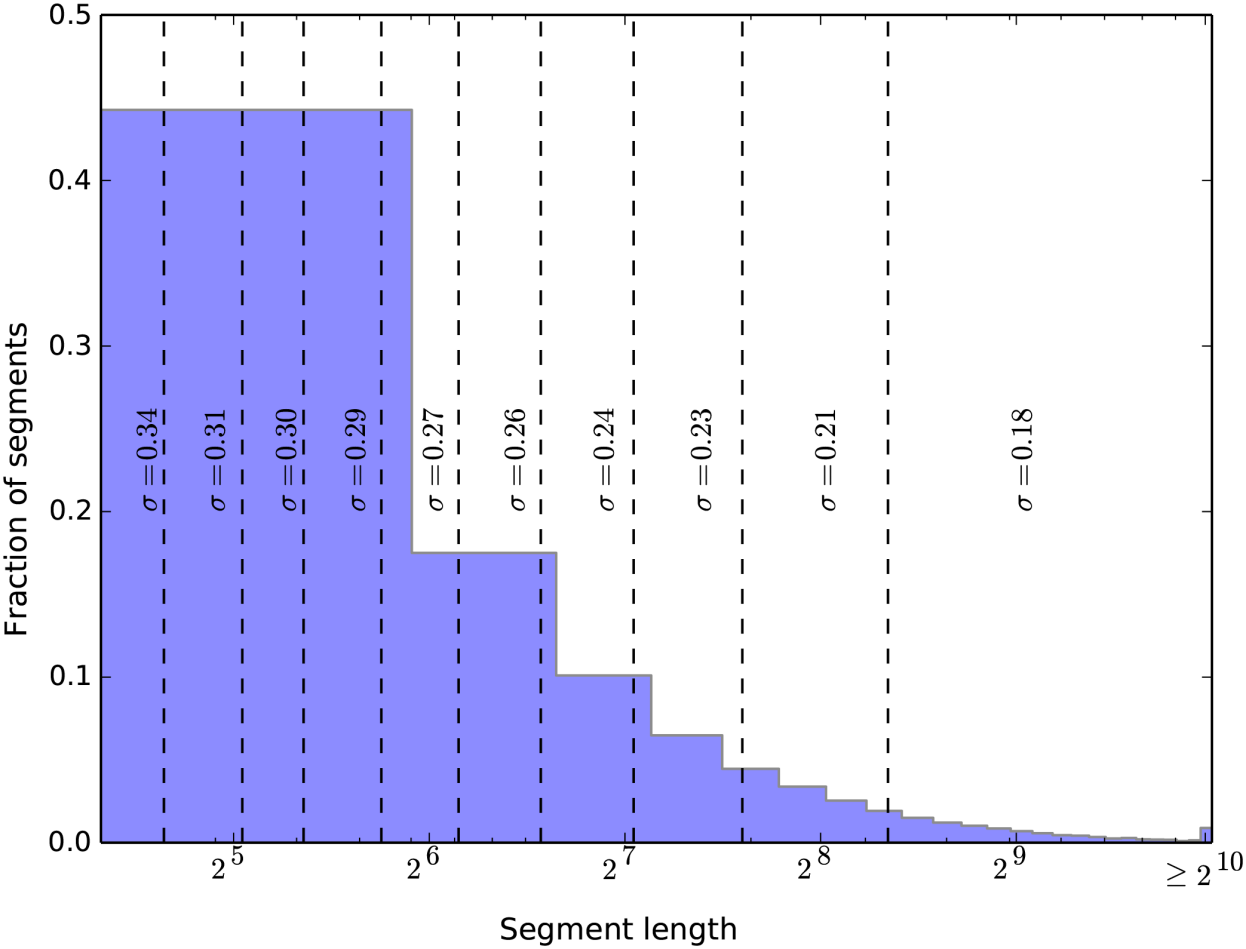
Segment length histogram overlaid with the shape parameters of the density ratio distributions for segment length groups (separated by dashed lines) shows that shorter segments tend to have more variable measurements. Segments were separated based on their length into 10 equal-content groups (group edges adjusted to allow for unique segment assignment), and the shape parameters *σ* were calculated from the inter-replicate errors of the measurements falling within each group (S1 Table).

Segment density ratios are estimates of the average number of ribosomes engaged in translation of a given segment (measured in arbitrary units), and are expected to become more reliable if the segment length is increased. Fig 3 shows that estimates obtained for longer segments are indeed more reliable (smaller *σ* values) with the longest segments (rightmost group) being nearly as reliable as the full-CDS estimates from all genes (S2 Fig). We note that although group widths increase almost exponentially, potentially collecting segments with different i.r.e. in the top group, the constructed groups map very well to individual levels of the segment trees because lengths of segments with each new level are halved on average. This mapping thus provides important additional information to the segment trees about the increasing reliability of measurements that are located higher within the tree.

**Fig 4:**
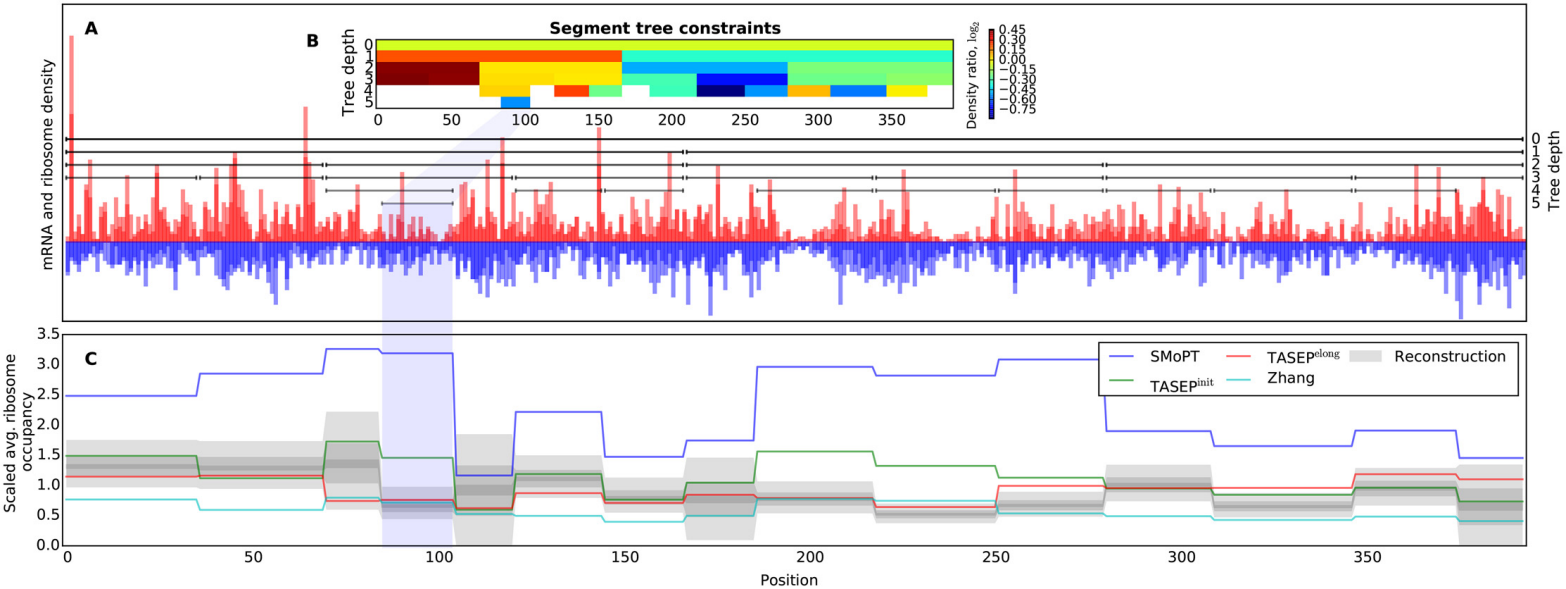
Ribosome profiling data, segment tree and simulated ribosome occupancy for gene YLR449W. (**A**) Ribo-seq (red) and mRNA-seq (blue) read density shown at single-codon resolution. Densities from the available replicates are overlaid with each other. Horizontal lines show the beginning and end of segments from the segment tree constructed for these densities. (**B**) Heatmap of the average density ratios recorded for each of the segments shows how density changes along the transcript for each of the scales (i.e. depths) within the segment tree. (**C**) Reconstruction of the per-transcript ribosome density from the segment tree (gray) shown as 90%, 50% and 10% confidence intervals (shades of grey). The reconstruction was obtained by sampling from the joint probability distribution derived from the segment tree (see S1 Text). Simulated ribosome occupancy for several considered models (blue, green, red and cyan solid lines) was averaged within segments and scaled to match the data.

In this way, segment trees establish a tradeoff between measurement reliability and measurement resolution by combining the use of trustworthy estimates high in the tree (corresponding to longer segments, describing high-level gene behavior) with the use of many less reliable estimates located lower in the tree that describe the local density variation. As can be seen from the visualization of the raw data for gene YLR449W and its segment tree reconstruction in Fig 4, our multi-scale approach, that combines measurements from different scales (segment lengths), allows for implicitly capturing changes in ribosome density along transcripts, while at the same time keeping the average ribosome density across larger segments close to the observed levels. This representation also encodes uncertainty about the density ratio at a particular region of the gene, even if that region is not directly represented by a segment from the tree. For example, region (85, 104) (highlighted in the figure) is covered by 6 segments (i.e. has depth 5 within the tree) and has one of the tightest confidence intervals (CIs) in the reconstruction. At the same time region (105, 120) was not measured at the two lowest scales (has a depth 3) and its average density has to be derived from the density values of other segments and our uncertainty about them, leading to a wider CI. This example demonstrates how segment trees capture changes in ribosome density along the transcript, which are crucial for fitting translation rates and evaluating competing models.

### Knowledge-based models do not fit RP data

Small standard deviations of the scaling factors and objective scores (determined using CV) of the evaluated models shown in Table 1 suggest that the (fitted) models perform consistently across different folds. The objective scores also show that knowledge-based models (i.e. the SMoPT and Zhang models) based on a manual choice of numerous translation-related parameters explain the ribosome density measurements significantly worse than the two models fitted on RP data. This can also be concluded from a visual inspection of the predictions made by these models for one of the genes in Fig 4C, which shows that their ribosome occupancies tend to “miss” the measured density ratios. For the Zhang model this could be explained by the absence of gene-specific initiation rates in the model, whereas SMoPT often overshoots the measured density ratios, presumably because it over-estimates initiation rates by neglecting ribosome queueing.

**Table 1:** Objective *ψ* and scaling factor *C* for the evaluated models computed on the test folds inside a 5-fold CV loop.

Model	Fitted	ln *C*	Objective *ψ*
Zhang	No	−4.55 ± 0.00	−600 286 ± 4449
SMoPT	No^†^	5.04 ± 0.01	−244 834 ± 2962
TASEP^init^	Init.	5.40 ± 0.00	99 144 ± 2137
TASEP^elong^	Yes	5.41 ± 0.02	114 865 ± 4335

^†^—RP data Ingolia *et al*. [10] was used in the original publication to set initiation rates.

**Fig 5:**
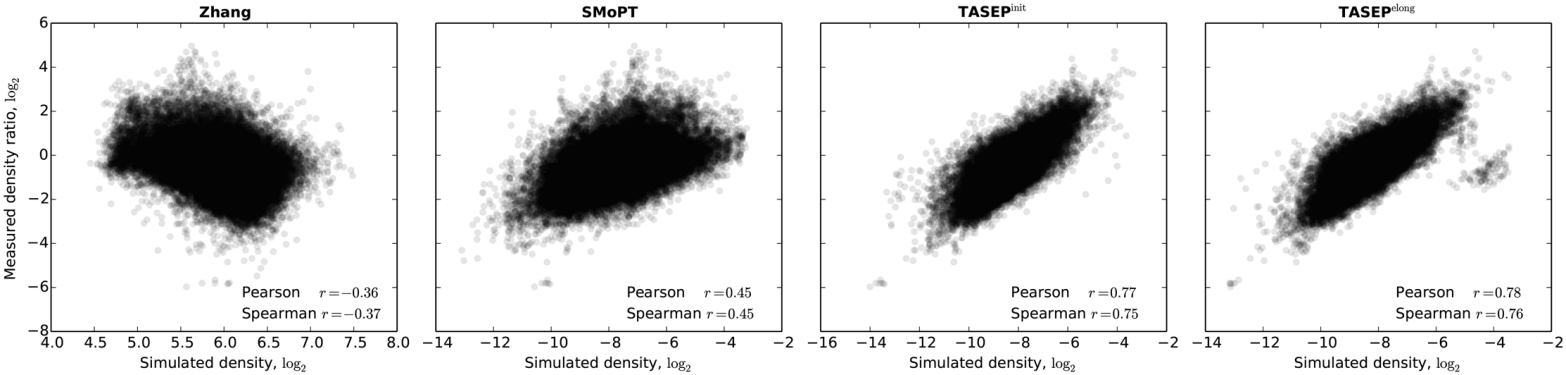
Measured segment density ratios *μ*_[*l*_*j*_,*r*_*j*_]_ plotted against the segment-averaged predicted ribosome occupancies for several existing and proposed models. Ribosome occupancy predictions made by the fitted models show significantly better agreement with the RP data. Reported correlations are highly significant (*p* < 10^−293^).

The TASEP^init^ model simulated with tAI-based elongation rates and fitted initiation rates achieves a significantly higher objective scores than the two state-of-the-art models. It is further improved by the TASEP^elong^ model, for which the elongation rates are additionally fit on the segment tree measurements. Fig 5 shows that superior objective function values of the fitted models translate to better predictions of the measured ribosome density (Pearson correlation coefficient *r* = 0.77 vs. 0.45, *p* < 10^−293^). Although the predictions are generally better for longer segments, improvements can be observed at all scales (see S3 Fig). While due to its relative simplicity only a weak positive correlation was expected for the Zhang model, for reasons unclear, a highly significant (*p* < 10^−293^) *negative* correlation is observed (Fig 5, left). This demonstrates that current knowledge-based models are not supported by RP measurements and highlights the importance of a critical evaluation of existing translation models against independent measurements.

### TASEP predictions are supported by independent datasets

Although TASEP^init^ and TASEP^elong^ outperformed existing models in the CV experiments, care has to be taken when interpreting these results as only the TASEP models were fitted directly on the segment tree measurements. We sought to obtain additional confirmation of the models’ performance and to determine if they make biologically meaningful predictions. To this end we compared the protein production and translation initiation rates given by TASEP models fitted on all segment tree estimates to several independent large-scale PA datasets (see Materials and Methods).

Most importantly, we found that for both models the predicted protein production rates (PPRs) *J* positively correlate with the PA measurements (Table 2). As expected, because *J* describes PPR per transcript, these correlations improve when the product of *J* and mRNA levels (*J* × mRNA; mRNA levels taken from the RP data) is considered. Even when both *J* and PAs are corrected for mRNA levels (thereby removing transcriptional regulatory influences in order to study translational regulation in isolation), the remaining (partial) correlation between *J*′ and PA′ is still significant, indicating that our TASEP models adequately capture the effects of protein translation on protein levels. These correlations are superior compared to correlations observed for state-of-the-art models (Table 3), especially when the partial correlations are considered. While strong positive partial correlations would be expected, we find these only for the fitted TASEP models. Unexpectedly low and negative partial correlations between PA′ and *J*′ for other models, together with strong correlations between PPR and mRNA levels (Table 4) suggest that existing models are overfit on transcript levels and may not accurately model translation. These findings provide an independent confirmation that the TASEP models with fitted translation rates accurately capture the dynamics of the *S. cerevisiae* translation machinery.

**Table 2:** Correlations of TASEP predictions with independent PA datasets. Spearman rank correlation coefficients *r* for are reported; *J*′ is the partial correlation between *J* and PA given mRNA.

	TASEP^init^		
	Newman YEPD	Newman SD	Ghaemmaghami
Init. rate	*r* = 0.56***	*r* = 0.55***	*r* = 0.49***
*J*	*r* = 0.57***	*r* = 0.56***	*r* = 0.50***
*J* × mRNA	*r* = 0.72***	*r* = 0.70***	*r* = 0.63***
*J*′	*r* = 0.52***	*r* = 0.49***	*r* = 0.39***
	TASEP^elong^		
	Newman YEPD	Newman SD	Ghaemmaghami
Init. rate	*r* = 0.54***	*r* = 0.53***	*r* = 0.49***
*J*	*r* = 0.56***	*r* = 0.53***	*r* = 0.49***
*J* × mRNA	*r* = 0.72***	*r* = 0.70***	*r* = 0.63***
*J*′	*r* = 0.52***	*r* = 0.48***	*r* = 0.39***

*—*p*–value < 10^−5^

**—*p*–value < 10^−20^

***—*p*–value < 10^−100^

**Table 3:** Correlations of predictions made by existing models with independent PA datasets. Spearman rank correlation coefficients *r* are reported.

	Siwiak and Zielenkiewicz		
	Newman YEPD	Newman SD	Ghaemmaghami
Init. rate	*r* = 0.45**	*r* = 0.48***	*r* = 0.40***
*J*	*r* = 0.33**	*r* = 0.36**	*r* = 0.37***
*J* × mRNA	*r* = 0.58***	*r* = 0.54***	*r* = 0.50***
*J*′	*r* = −0.12*	*r* = −0.07	*r* = −0.01
	SMoPT		
	Newman YEPD	Newman SD	Ghaemmaghami
Init. rate	*r* = 0.45**	*r* = 0.49***	*r* = 0.44***
*J*	*r* = 0.21**	*r* = 0.23**	*r* = 0.26**
*J* × mRNA	*r* = 0.45**	*r* = 0.46**	*r* = 0.46***
*J*′	*r* = −0.26**	*r* = −0.21*	*r* = −0.13*
	Ciandrini *et al*. [21]		
	Newman YEPD	Newman SD	Ghaemmaghami
Init. rate	*r* = 0.44***	*r* = 0.43***	*r* = 0.43***
*J*	*r* = 0.45***	*r* = 0.44***	*r* = 0.44***
*J* × mRNA	*r* = 0.57***	*r* = 0.56***	*r* = 0.55***
*J*′	*r* = 0.10*	*r* = 0.10*	*r* = 0.14*

*—*p*–value < 10^−5^

**—*p*–value < 10^−20^

***—*p*–value < 10^−100^

**Table 4:** Comparison of TASEP predictions to existing models. Spearman rank correlation coefficients *r* are reported. When “corrected for” column is non-empty, partial correlations are reported.

Variable 1	Variable 2	Corrected for	Correlation coeff.	*p*-value
TASEP^init^ init. rates	SMoPT init. rates		*r* = 0.67	*p* < 10^−298^
	Siwiak and Zielenkiewicz init. rates		*r* = 0.74	*p* < 10^−298^
	Ciandrini *et al*. init. rates		*r* = 0.47	*p* < 10^−197^
TASEP^elong^ init. rates	TASEP^init^ init. rates		*r* = 0.94	*p* < 10^−298^
	SMoPT init. rates		*r* = 0.65	*p* < 10^−298^
	Siwiak and Zielenkiewicz init. rates		*r* = 0.73	*p* < 10^−298^
	Ciandrini *et al*. init. rates		*r* = 0.46	*p* < 10^−182^
CDS lengths	TASEP^init^ init. rates		*r* = −0.07	*p* < 10^−4^
	TASEP^elong^ init. rates		*r* = −0.05	*p* < 10^−2^
	SMoPT init. rates		*r* = −0.52	*p* < 10^−257^
	Siwiak and Zielenkiewicz init. rates		*r* = −0.02	*p* > 10^−1^
	Ciandrini *et al*. init. rates		*r* = −0.65	*p* < 10^−298^
5′ UTR lengths	TASEP^init^ init. rates		*r* = −0.01	*p* > 10^−1^
	TASEP^elong^ init. rates		*r* = −0.02	*p* > 10^−1^
	SMoPT init. rates		*r* = −0.06	*p* < 10^−3^
	Siwiak and Zielenkiewicz init. rates		*r* = 0.00	*p* > 10^−1^
	Ciandrini *et al*. init. rates		*r* = −0.09	*p* < 10^−10^
	TASEP^init^ init. rates	CDS lengths	*r* = 0.00	*p* > 10^−1^
	TASEP^elong^ init. rates	CDS lengths	*r* = −0.01	*p* > 10^−1^
	SMoPT init. rates	CDS lengths	*r* = 0.03	*p* > 10^−1^
	Siwiak and Zielenkiewicz init. rates	CDS lengths	*r* = 0.03	*p* < 10^−1^
	Ciandrini *et al*. init. rates	CDS lengths	*r* = −0.06	*p* < 10^−3^
3′ UTR lengths	TASEP^init^ init. rates		*r* = 0.04	*p* < 10^−2^
	TASEP^elong^ init. rates		*r* = 0.04	*p* < 10^−1^
	SMoPT init. rates		*r* = 0.06	*p* < 10^−3^
	Siwiak and Zielenkiewicz init. rates		*r* = 0.07	*p* < 10^−5^
	Ciandrini *et al*. init. rates		*r* = 0.03	*p* < 10^−1^
	TASEP^init^ init. rates	CDS lengths	*r* = 0.04	*p* < 10^−1^
	TASEP^elong^ init. rates	CDS lengths	*r* = 0.04	*p* < 10^−1^
	SMoPT init. rates	CDS lengths	*r* = 0.07	*p* < 10^−4^
	Siwiak and Zielenkiewicz init. rates	CDS lengths	*r* = 0.08	*p* < 10^−6^
	Ciandrini *et al*. init. rates	CDS lengths	*r* = 0.02	*p* > 10^−1^
mRNA levels	TASEP^init^ init. rates		*r* = 0.36	*p* < 10^−115^
	TASEP^elong^ init. rates		*r* = 0.33	*p* < 10^−93^
	SMoPT init. rates		*r* = 0.58	*p* < 10^−298^
	Siwiak and Zielenkiewicz init. rates		*r* = 0.33	*p* < 10^−117^
	Ciandrini *et al*. init. rates		*r* = 0.62	*p* < 10^−298^
	TASEP^init^ *J*		*r* = 0.34	*p* < 10^−97^
	TASEP^elong^ *J*		*r* = 0.37	*p* < 10^−115^
	SMoPT *J*		*r* = 0.65	*p* < 10^−298^
	Siwiak and Zielenkiewicz *J*		*r* = 0.69	*p* < 10^−298^
	Ciandrini *et al*.*J*		*r* = 0.63	*p* < 10^−298^
mRNA levels	Newman YEPD PA		*r* = 0.58	*p* < 10^−209^
	Newman SD PA		*r* = 0.57	*p* < 10^−194^
	Ghaemmaghami PA		*r* = 0.54	*p* < 10^−273^
CDS lengths	Newman YEPD PA		*r* = −0.13	*p* < 10^−10^
	Newman SD PA		*r* = −0.14	*p* < 10^−12^
	Ghaemmaghami PA		*r* = −0.16	*p* < 10^−22^
	Newman YEPD PA	mRNA	*r* = 0.32	*p* < 10^−60^
	Newman SD PA	mRNA	*r* = 0.28	*p* < 10^−42^
	Ghaemmaghami PA	mRNA	*r* = 0.21	*p* < 10^−36^
	mRNA		*r* = −0.53	*p* < 10^−298^
	5′ UTR lengths		*r* = 0.14	*p* < 10^−20^
	3′ UTR lengths		*r* = −0.03	*p* < 10^−1^

Looking more in detail (Table 4), we find that for both models the fitted initiation rates agree well with the rates inferred by the existing full-cell models of Shah *et al*. (SMoPT), and of Siwiak and Zielenkiewicz. However, we did not find the previously reported strong negative correlation between initiation rates and CDS length [9, 21]. We note that this correlation is also not supported by the model of Siwiak and Zielenkiewicz. The initiation rates also exhibit a weak correlation with the 3′ UTR lengths (similar correlations also found for several other models), supporting the hypothesis of more efficient translation re-initiation in genes with longer 3′ UTRs.

Interestingly, we did not find the tendency for genes with short 5′ UTRs to exhibit high initiation rates suggested by Shah *et al*. and supported by Ciandrini *et al*. [21] in our models or the model of Siwiak and Zielenkiewicz. We also note that no relationship or a negative relationship can be observed between initiation rates and 5′ UTR lengths corrected for CDS lengths can be found in most considered models. This suggests that the previously observed inverse relationship between 5′ UTR lengths and initiation rates may not be indicative of a 5′ UTR-mediated initiation rate regulation mechanism, but could be merely a consequence of a positive correlation between 5′ UTR lengths and CDS lengths.

While correlations observed for the fitted models do not change between TASEP^init^ and TASEP^elong^ (Table 4), the latter model makes considerably better ribosome occupancy predictions. It can be seen from the example in Fig 4C that fitting the elongation rates allows the segment-averaged ribosome occupancy of TASEP^elong^ to follow the reconstructed density considerably better than any of other model.

### Fitted elongation rates are not explained by adaptation to tRNA levels alone

Since the TASEP^elong^ model achieves a significantly better fit to the RP data compared to TASEP^init^ with tAI-based rates (Table 1), having fitted its elongation rates on different CV folds, we sought to interpret the obtained values and their variance. We first, however, confirmed that elongation rates determined from different RP datasets agree qualitatively with each other by fitting a new TASEP^elong^ model on the dataset of Ingolia *et al*. [10] and comparing its translation rates to the original model (see S1 Text).

**Fig 6:**
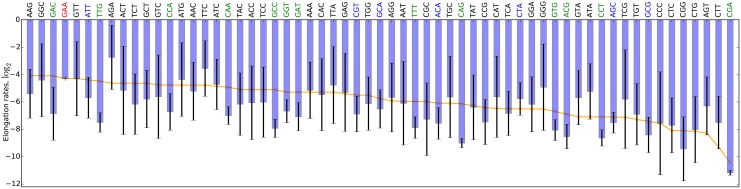
Mean and SD of the codon elongation rates fitted in different CV folds, compared to the tAI-based rates. For many codons elongation rates (depicted as mean and SD, blue bars) are reproducible across CV folds. This becomes evident for codons with smaller SDs (blue labels, *σ* < 1.5), and codons whose elongation rates are significantly different from the tAI-based rates (green labels; *t*-test, *p* < 0.05). tAI-based rates (orange line) are plotted as a reference. The rate of codon GAA (red label) was not optimized. Stop codons were excluded from the figure as their fitted termination rates remained very close to the original values of 1.

It can be seen from Fig 6 that despite the generally large SDs, for many codons the elongation rates fitted in different folds of the CV are spread compactly around codon-specific values. This is clearly visible for codons with smaller SDs (green and blue), for which similar rates were found in different folds. Nonetheless the rate SDs differ considerably between codons. While the majority of the fitted elongation rates are consistently different from tAI-based rates, only for 13 codons this difference is statistically significant (single sample *t*-test for population mean difference, *p* < 0.05; Fig 6, S2 Table): GAC, TTG, CCA, CAA, GCC, GGT, GAT, TTT, CAG, GTG, ACG, CCT and CGA. Although these differences between the tAI-based and fitted elongation rates are challenging to explain, their presence suggests that additional unknown factors are shaping these rates.

**Fig 7:**
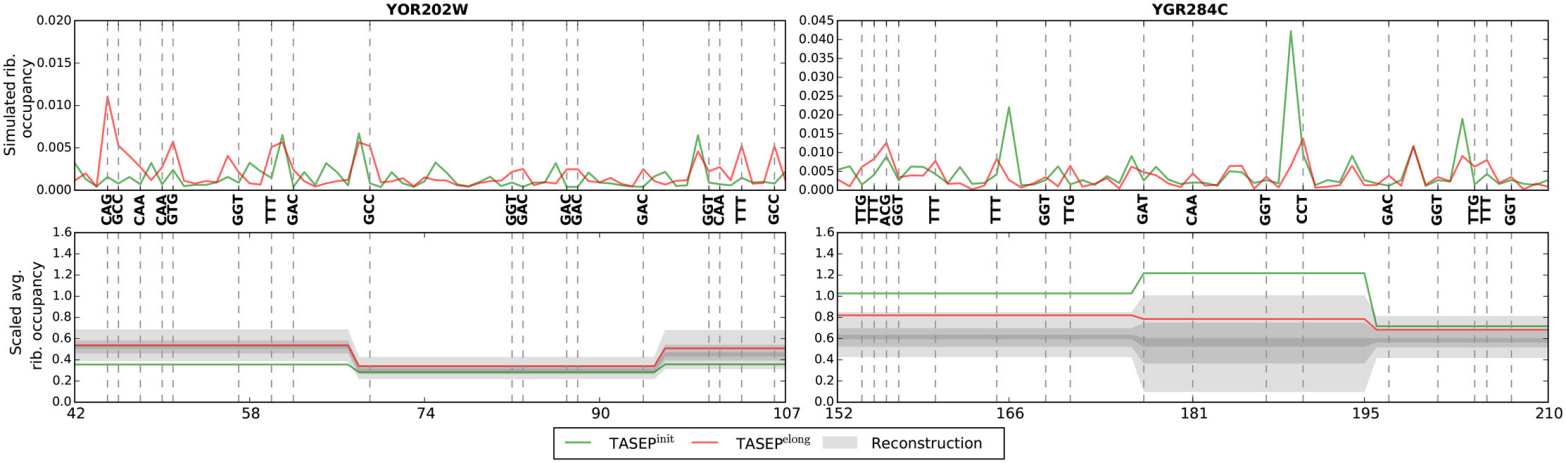
Ribosome density reconstruction (gray, bottom panels) and simulated ribosome occupancy (top) for selected regions of genes YOR202W (left panels) and YGR284C (right panels) plotted for the TASEP^init^ (green) and TASEP^elong^ (red) models. Presence of codons with significantly different elongation rates (vertical dashed lines) increases simulated ribosome occupancy. Higher increase can be observed for segments containing more such codons. This is clearly seen for gene YOR202W (left) with similar initiation rates in the TASEP^init^ and TASEP^elong^ models (0.24 × 10^−4^ and 0.22 × 10^−4^ respectively), for which the predicted occupancy only increases when fitted elongation rates are used. For most genes, such as YGR284C (right) this increase in density is compensated by reducing the initiation rate (from 0.72 × 10^−4^ to 0.36 × 10^−4^), which leads to an overall better agreement between simulated ribosome occupancy and the segment tree measurements (bottom right). To keep the visualization comprehensible, only selected regions of genes YOR202W and YGR284C were used. However, the described trends also hold for the remainder of these genes and for other genes.

Having identified differences in elongation rates between the TASEP^init^ and TASEP^elong^ models, we sought to understand their effect on models’ predictions. As could be expected from the similar correlations in Table 4 and Fig 5, the two models make very similar PPR and ribosome density predictions (S4 Fig). However, ribosome density predicted by the TASEP^elong^ model with fitted elongation rates agrees better with RP measurements. To understand the cause of this improvement we looked for genes whose fit to the RP data improved when fitted elongation rates were used. These genes can be classified into two groups: (i) genes that have a very similar initiation rate in both models (Fig 7, left); and (ii) genes that have a considerably lower initiation rate in the TASEP^elong^ model (Fig 7, right). Because all 13 codons with significantly different elongation rates were predicted to be slower, their presence in CDSes generally leads to higher predicted ribosome occupancy, especially if the genes initiation rate remains unchanged. For genes from the first group, such as YOR202W shown on the left panel of Fig 7, this already results in a more accurate ribosome occupancy prediction. For most other genes, the second group, this increase in codon elongation times yields ribosome occupancy that is too high under the current initiation rate. For these genes (e.g. YGR284C on the right panel of Fig 7) a smaller fitted initiation rate is required to reduce ribosome occupancy that would otherwise be too high due to the effects of slow codons and high ribosomal flux (due to high initiation rate). Together these effects allow the model to better match the ribosome density changes along the transcript.

### Significance of the fitted elongation rates for codon optimization

Codon optimization, the process of substituting codons with synonymous alternatives that are elongated faster, thus contributing to the overall protein production rate, is routinely used to improve protein expression [39, 40]. Nonetheless, it remains a controversial tool because the same optimization techniques can lead to contradicting results when applied to different proteins [41]. Here we compare our fitted elongation rates to codon optimality estimated by the commonly used tAI [32] and CAI [34] indices.

**Fig 8:**
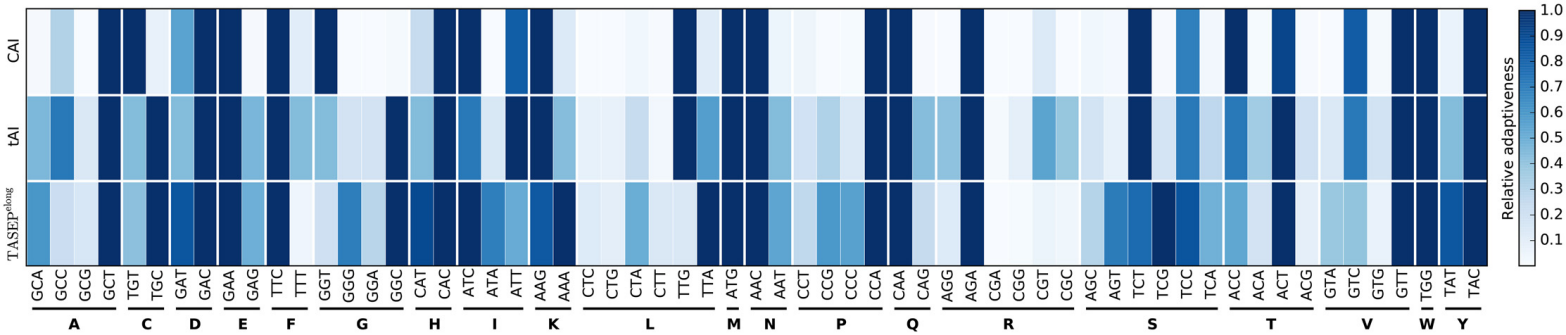
tAI and CAI compared to a measure of codon adaptation derived from the fitted TASEP^elong^ elongation rates. Relative adaptiveness of codons grouped by their corresponding amino acids (columns) plotted for the three measures of codon adaptation (rows) shows that the considered measures often agree on the optimal codon. In particular, the tAI and TASEP^elong^ measure agree on the optimal codons for all but 4 amino acids (I, K, L and S).

We considered the relative adaptiveness of a codon (see Materials and Methods) given by the CAI, the tAI and the fitted elongation rates of the TASEP^elong^ model. Fig 8 shows that the three measures of codon adaptation often agree on the optimal codon for a particular amino acid (relative adaptiveness of 1.0, dark blue), which further demonstrates that our findings are in line with the earlier work. In particular, despite significant differences between the fitted elongation rates and elongation rates given by the tRNA adaptation hypothesis, the two sets agree on optimal codons for all but four amino acids. Only for isoleucine, leucine, lysine and serine the TASEP^elong^ model suggests codons ATC, AAA, TTA and TCG instead of ATT, AAG, TTG and TCT respectively. An interesting observation is that the bottom row in Fig 8 is much more blue than the top ones, suggesting codon optimization is less black-and-white than suggested by tAI and in particular CAI, meaning that many more codons are “reasonably good”, i.e. there may be less to gain by codon optimization than thought before. This observation is also corroborated by Leavitt and Alper [42], who noted that the level of control achievable in yeast through codon optimization is considerably smaller than what can be achieved through transcriptional regulation.

### Translation initiation limits protein production

It is still unclear whether translation of endogenous yeast genes is limited by initiation or elongation [43, 44]. To test whether translation is limited by the initiation rates or by the elongation rates we artificially increased the initiation rate of each gene from the TASEP^elong^ model by 10%. To obtain robust results the experiment was repeated 5 times with different random initializations and the average increase in PPRs was calculated for every gene.

**Fig 9:**
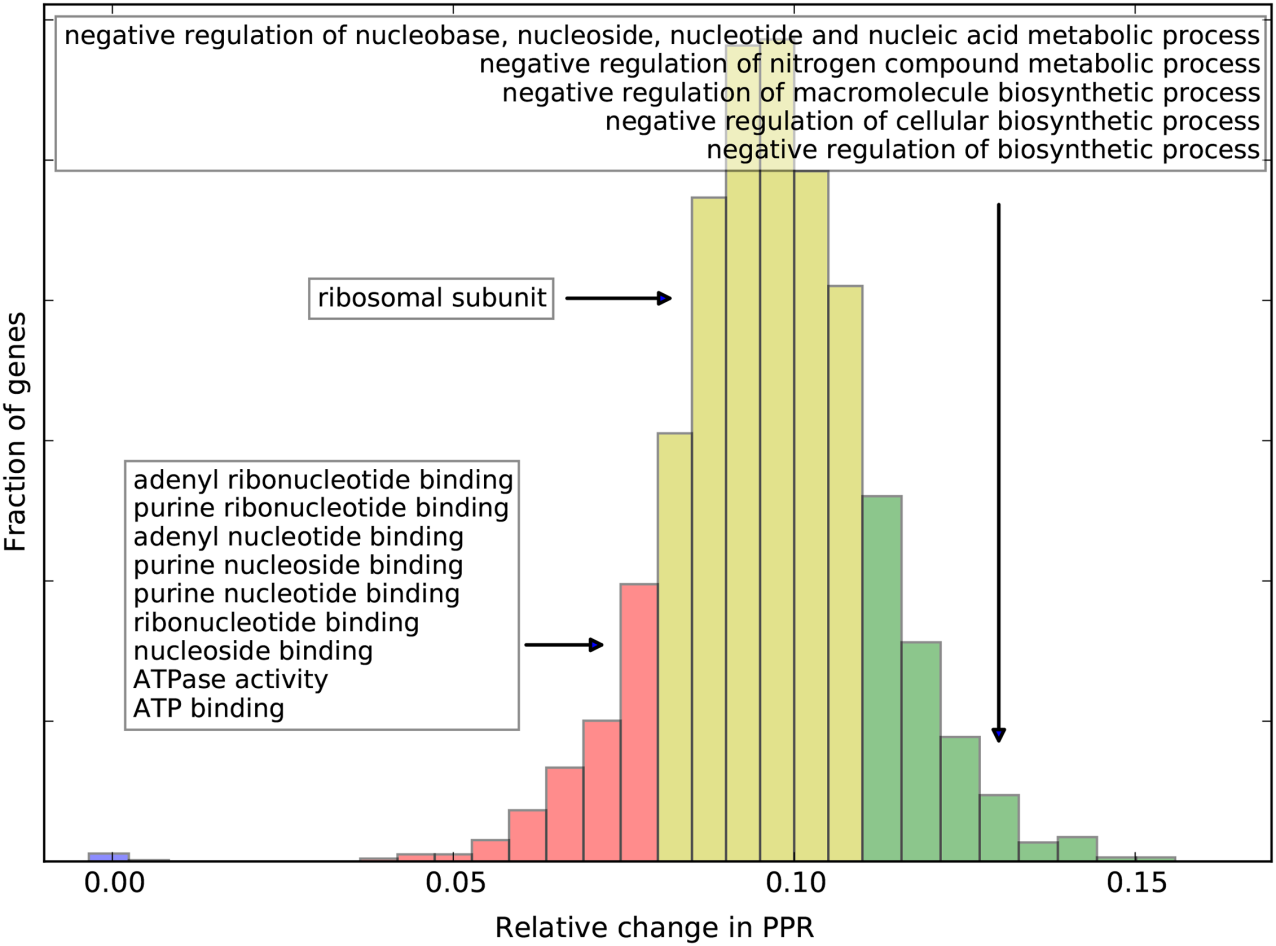
Distribution of the relative changes in PPR after a 10% increase in initiation rates shows that translation initiation is the rate limiting step for the protein production for most *S. cerevisiae* genes from the considered RP dataset. Groups of genes with low (≥ 0.02 and < 0.08, red), medium (≥ 0.08 and < 0.11, yellow) and high (≥ 011, green) increase in PPRs are enriched for several biological functions (white boxes in the figure, FDR < 0.05).

Fig 9 shows the relative differences in PPRs for all genes. In almost all cases (except 7 genes) the PPR increased substantially (relative difference > 0.02) when increasing the initiation rate, supporting the hypothesis that under exponential growth in the rich medium translation in *S. cerevisiae* operates in an initiation-limited regime. This also explains why fitting the codon elongation rates in TASEP^elong^ did not improve the PA correlations compared to the TASEP^init^ model. Elongation-limited production for these genes can be explained by the very high initiation rates predicted for them, which shift the rate-limiting step from translation initiation to translation elongation. Interestingly, groups of genes that had a low, medium and high PPR increase are enriched for several biological functions (FDR < 0.05, Fig 9). Notably, genes in the high increase group are involved in negative regulation of various biosynthetic and metabolic processes. This suggests that yeast cells may have evolved to rapidly “switch on” negative regulation by keeping a buffer of the required mRNA transcripts that are efficiently translated only once there is demand.

## Discussion

For the first time, we described an approach that derives complete translation kinetics of an organism from ribosome profiling data and used it to simultaneously infer the translation elongation, translation initiation and protein production rates all together without neglecting the effects of ribosomal interference. We applied our methodology to the ribosome and RNA sequencing data of the baker’s yeast *Saccharomyces cerevisiae*. The fitted yeast translation models agree considerably better with independent protein abundance datasets than existing models. In particular, our TASEP models are the only ones that maintain strong correlations with protein abundance after removing the effect of transcriptional regulation.

While translation initiation rates provided by the models are similar to rates from other studies, we did not find the previously reported negative correlation between initiation rates and CDS lengths. The observed negative correlations between PA and CDS length, which one would expect to see as a result of this correlation, can alternatively be explained by transcriptional regulation, i.e. the strong negative correlation between mRNA levels and CDS lengths (Table 4). An alternative explanation can be offered by a mechanism driven by amino acid chain elongation rather than translation initiation. For example, abortive translation or the degradation of misfolded proteins [45], since the chance of producing a misfolded protein is expected to increase with protein length.

We also found that translation elongation rates deviate considerably from the widely accepted tRNA pool adaptation hypothesis, for 13 codons significantly so. Differences in elongation rates of these codons between the tRNA pool adaptation hypothesis and TASEP^elong^ may be partially explained by nucleotide modifications of their respective tRNAs, which are known to modify the specificity and efficiency of messenger decoding [46]. As such, some of these 13 codons were shown to be affected by post-transcriptional nucleotide modifications of tRNAs in different organisms [47]. We speculate that for these codons the concentration of (un)modified tRNAs, rather than the total tRNA concentration, plays a non-negligible role in determining their elongation rates [18]. An additional factor that possibly contributes to the observed deviation from the tRNA pool adaptation hypothesis is its implicit assumption that different tRNA genes from the same family contribute equally to determining the rate of translation. This assumption should be revisited in light of the recent finding of Bloom-Ackermann *et al*. [48] that the contributions of different gene copies from the same tRNA family to the tRNA pool and cellular fitness are far from equal.

In our experiments we found that SDs of elongation rates from different CV folds differ markedly between codons. In order for the elongation rates to be specified with high precision by the RP data, small changes in the rates must lead to detectable differences in ribosome density. However, in light of our finding that yeast translation is initiation-limited and the observation of Bloom-Ackermann *et al*. [48] that *S. cerevisiae* is robust to deletions of tRNA genes, especially in rich medium used to produce the ribosome profiling measurements analyzed here, it is unlikely that in the considered physiological conditions the elongation rates exert a strong enough effect on ribosome density to allow the RP data to specify elongation rates with high precision. We speculate that found SDs reflect the robustness of the yeast translation system w.r.t. the codon translation rates, with the system being more sensitive to changes in rates of those codons that have smaller SDs. In this case, yeast translation appears to be robust to fold changes in codon translation rates and, consequently, to the aminoacyl-tRNA availability that these rates are thought to be determined by [44].

Alternatively, the SDs may reflect the extent to which codon translation rates change between CV folds due to codon context, i.e. the local sequence around a codon which may alter its elongation rate (see S1 Text, translation rate reproducibility analysis). It is unlikely that the TASEP model captures the full complexity of the translation process by assuming that codon elongation rates are determined solely by the codon identity, and not also by the sequence surrounding the codons as was previously suggested [2, 3]. Such a constraint limits the models ability to capture the underlying translation dynamics and may bias it towards fitting different rates on different sets of genes (e.g. CV folds) with varying codon contexts, thereby inflating the SDs. The observed variation in fitted elongation rates puts forward codon context as a factor that may significantly modulate the baseline elongation rates.

Using our models we found that under exponential growth in rich medium translation initiation appears to be the main limiting factor of protein production of endogenous genes in *Saccharomyces cerevisiae*, with protein production being limited by initiation rates for all but 7 genes with very high initiation rates. These findings suggest that rational design of 5′ UTRs involved in translation initiation [49, 50] may be a more promising avenue for achieving protein overexpression than the routinely used codon optimization techniques. It is likely, however, that further overexpression could be achieved using codon optimization. Because once the gene is put under the translational control of an efficient 5′-UTR, which is usually the case in heterologous gene expression, translation elongation is expected to become a rate-limiting factor. In such cases we recommend performing codon optimization using the fitted TASEP^elong^ elongation rates, which, while mostly agreeing with existing techniques, also demonstrate several differences.

Although we found that translation initiation appears to be the main factor limiting protein production in yeast under exponential growth in rich medium, it is possible that different mechanisms are dominant in other organisms. For example, Li *et al*. [51] and Guimaraes *et al*. [52] discuss greater contribution of protein elongation respectively by anti-Shine-Dalgarno sequences and codon usage in *E. coli*. Our method could be applied to ribosome profiling data of other organisms to delineate the relative contribution of initiation and elongation.

All translation models proposed to date, including TASEP^init^ and TASEP^elong^, assume that translation elongation rates are not influenced by *codon context*, i.e. the sequence around a particular codon, although various factors affecting the speed of elongation have been suggested [2–4]. Variation in fitted elongation rates and the highly varying codon translation times recently observed by Dana and Tuller [53] suggest that codon context may play a more compelling role in determining translation rates than previously thought. Fortunately investigations of codon context are becoming feasible thanks to the growing adoption of ribosome profiling as a standard technique for studying translation. With the increasing amount of ribosome profiling measurements, data-driven approaches, such as the one described here, will become instrumental for delineating the effects of multiple competing translation mechanisms, for generating new hypothesis, and for constructing predictive models for use in other fields. These goals can be achieved by incorporating alternative translation mechanisms as sequence- and position-specific effects altering the codon elongation rates.

## Supporting Information

S1 Text Contains extended methods and supplementary results. (PDF)

S1 Fig Histograms of the log_2_ inter-replicate errors (ratios of replicated measurements) of reliable ribosome and mRNA density measurements show that the full-CDS and segment tree density estimates follow comparable log-normal distributions. Distributions fitted into data (solid lines) are centered around zero, but their SDs differ.
(TIF)

S2 FigHistograms of the log_2_ inter-replicate errors of reliable density ratio measurements show similar error profiles in full-CDS and segment tree estimates.The group shape parameters of the i.r.e. and the density ratio distributions are related as $\sigma_{k}^{\text{group}}=\frac{1}{\sqrt{2}}\sigma_{k}^{\text{i.r.e.}}$.(TIF)

S3 Fig Measured segment density ratios *μ*_[*l*_*j*_,*r*_*j*_]_ plotted against the segment-averaged predicted ribosome occupancies for segments of varying size and for several existing and proposed models. TASEP^init^ and TASEP^elong^ significantly improve over existing models for all segment length groups.
(TIF)

S4 Fig Agreement between the PPR (left) and gene-level average ribosome occupancy (right) predictions made by TASEP^init^ and TASEP^elong^ models. (TIF)

S5 Fig Presence of codons in gene and segment sequences from the segment tree. Translation rate of codon GAA (red) was fixed in elongation rate fitting experiments as it is present in many genes and segments.
(TIF)

S6 Fig Histogram of the running times (average over 3 replicates) of the TASEP model simulations for genes in the evaluation set. tAI-based elongation rates and initiation rates of 1.0 were used in the simulations.
(TIF)

S1 Table Shape parameters of the density ratio distributions for segments grouped by length. Left (inclusive) and right (exclusive) edges give the range of segment lengths of a given group.
(PDF)

S2 Table Mean and SD of the codon elongation rates fitted on different CV folds of the evaluation set. *p*-values of the single sample *t*-test are calculated to check wether the observed rates are significantly different from the tAI-based rates. All rates are given in log_2_ space. Codons are colored as in the main text.
(XLS)

S3 Table Detailed results of the GO term functional enrichment analysis. (XLS)

S1 Dataset Translation initiation and protein production rates for the derived models. (CSV)
