# Supplementary material for: Unbiased Quantitative Models of Protein Translation Derived from Ribosome Profiling Data
Source: PLoS Comput Biol. 2015 Aug 14;11(8):e1004336. doi: 10.1371/journal.pcbi.1004336 (PMC4537299; doi:10.1371/journal.pcbi.1004336)
Supplement: S1 Text — (PDF) [file pcbi.1004336.s001.pdf]

Supplementary Information for "Deriving unbiased quantitative models of protein translation from ribosome profiling data"

Alexey A. Gritsenko [1,3,4], Marc Hulsman [1], Marcel J.T. Reinders [1,4], Dick de Ridder [1,2,3,4,*]
1 The Delft Bioinformatics Lab, Department of Intelligent Systems, Delft University of Technology, Mekelweg 4, 2628 CD Delft, The Netherlands
2 Bioinformatics Group, Wageningen University, Droevendaalsesteeg 1, 6708 PB, Wageningen, The Netherlands
3 Platform Green Synthetic Biology, P.O. Box 5057, 2600 GA Delft, The Netherlands
4 Kluyver Centre for Genomics of Industrial Fermentation, P.O. Box 5057, 2600 GA Delft, The Netherlands
∗ E-mail: dick.deridder@wur.nl

SI-1 Ribosome profiling read processing

With the exception of elongation rate reproducibility analysis, the RP data for yeast *Saccharomyces cerevisiae* strain S288C [1] were used for all analyses. These data are available as raw sequencing reads that needed to be trimmed and aligned to the genome prior to any analysis. The read mapping procedure from [2] was used to align RP reads to the yeast genome. After trimming the reads to a length of 21 nt to remove any linker-adapter sequences, the trimmed reads were aligned to the S288C reference genome sequences (release R64, accessed on January 14, 2014) using Bowtie [3]. First, the reads were mapped to the annotated CDSes and UTRs (taken from SGD [4]) of S288C extended by 100 nt on each side, and then unaligned reads were mapped to the entire reference genome sequence. These alignments were then extended upto the original read length to minimize the number mismatches between the untrimmed read, the reference and the linker sequences. Sequences `CTGTAGGCACCATCAAT` and `AGATCGGAAGAGCACACGTCTGA` were used for the RP linker and Illumina sequencing adapter during extension. Alignments with up to 2 mismatches were accepted, and multiple alignments were allowed for a single read, but alignments with fewer mismatches were preferred. Following McManus *et al.* [1] only RNA- and ribo-seq alignments of lengths $27 \leq l \leq 40$ and $27 \leq l \leq 33$ respectively were kept for analysis.

We then sought to assign reads to the (parts of) CDSes that they originate from. Ribo-seq reads should be assigned to CDSes based on the position of the A-site codon in the read, which may differ with read length. Metagene analysis [2] was used to calibrate the position of the A-site codon for various footprint lengths. Reads with alignments containing start or stop codons of annotated CDSes were considered and the positions of these codons were recorded. Histograms of the positions of the start and stop codons in ~~Figure~~Fig SI-1 were then used to determine the location of the P-site for each footprint length. The footprints were then assigned to CDSes based on the alignment coordinate of the overlap of the second nucleotide of the A-site codon (i.e. P-site offset +4) with annotated CDSes. RNA-seq reads were similarly assigned to CDSes based on the coordinate of their central nucleotide. For reads that map to multiple locations (ambiguous reads) an equal fraction of the read count was counted towards each location; and reads assigned to multiple CDSes ("overlapping" reads) contributed their full read count to each assignment region. Read assignment was performed separately for each of the two mRNA- and ribo-seq biological replicates (see Table SI-2 for statistics).

Table SI-1. P-site offsets for various footprint lengths determined individually for each replicate based on ~~Figure~~Fig SI-1.

Length	Replicate 1		Replicate 2		Final offset
	Start	Stop	Start	Stop	
27	12	11	11	11	11
28	12	12	12	12	12
29	13	13	13	13	13
30	13	13	14	14	14
31	14	14	14	14	14
32	14	14	14	14	14
33	14	14	14	14	14

Table SI-2. Read alignment statistics for the *S. cerevisiae* genome release R64, and varying alignment strictness. Counts for reads aligned to any position in the reference genome and counts for reads assigned to coding sequences are reported separately; CDS counts were rounded to the nearest integer (in cases when reads had multiple alignments, only the fraction of alignments assigned to CDSes was counted).

Type	Name	Max. subst.	Min. length	Max. length	Unique	Replicate 1		Replicate 2	
						mRNA	Ribosome	mRNA	Ribosome
Ref.	Filtered	2	27	33/40	No	13,504,666	19,990,853	13,567,103	22,766,209
	Strict	1	27	33/40	No	12,827,218	18,782,657	12,870,000	21,360,880
	Unique	1	27	33/40	Yes	11,468,200	15,962,773	11,534,794	17,796,356
CDS	Filtered	2	27	33/40	No	10,859,192	15,983,167	10,911,163	16,173,551
	Strict	1	27	33/40	No	10,314,950	15,072,767	10,350,534	15,300,543
	Unique	1	27	33/40	Yes	9,037,207	12,936,748	9,081,107	12,945,505

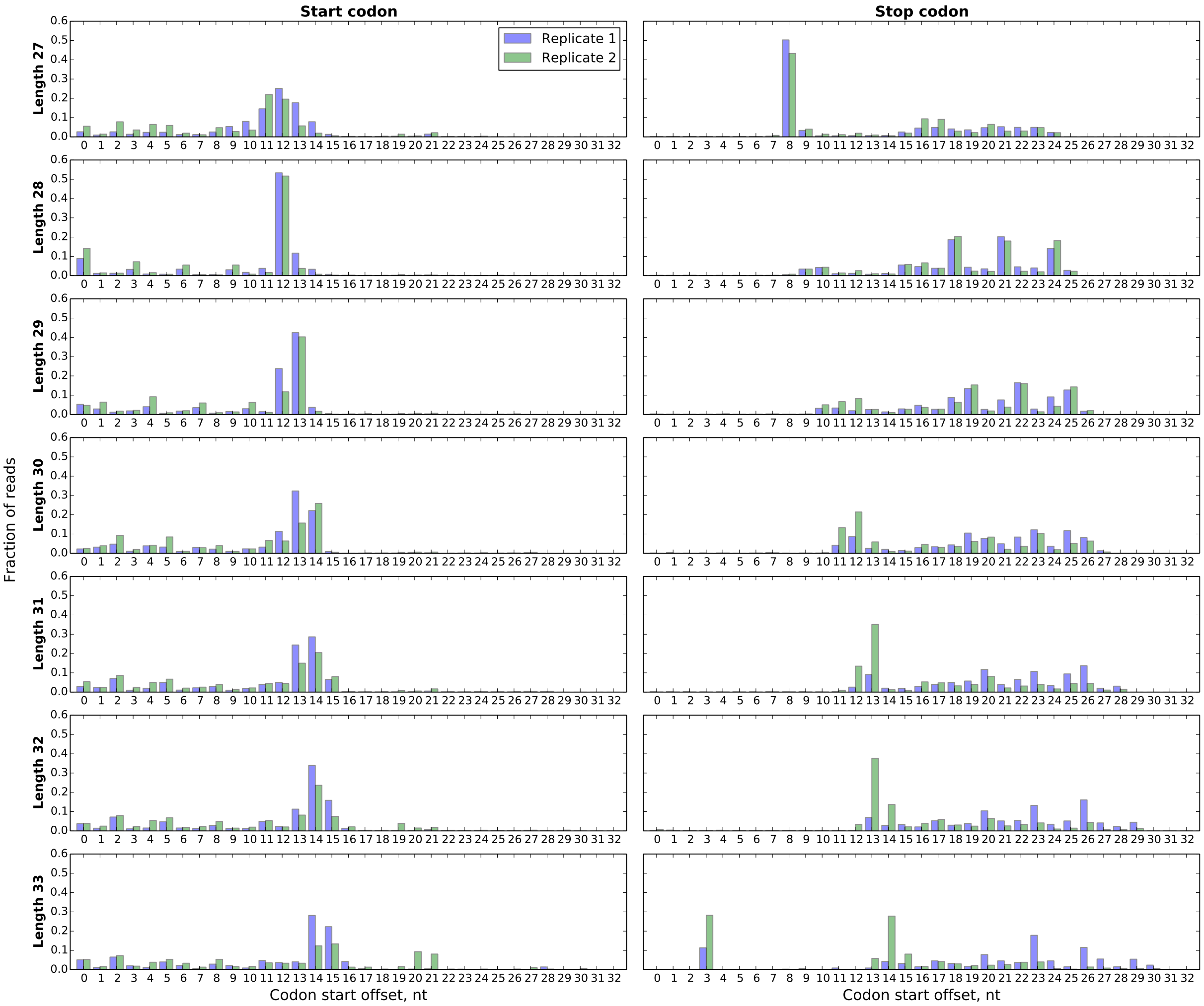

Figure SI-1. Histograms of the locations of the start and stop codons within ribosomal footprints of various lengths. Offsets give distances from the beginning of the read to the first nucleotides of the start or stop codons. High peaks around positions 11-14 on the start codon offset histograms directly give position of the P-site in ribosomal footprints, whereas the leftmost periodic peaks in stop codon offset histograms are located 6 nt upstream of the P-site. Offsets fixed for every footprint length are given in Table SI-1.

SI-2 Setting the read count thresholds

MA-plots typically applied to the analysis and normalization of 2-channel microarray data [5] were used to visualize density measurement differences between replicates by plotting the log-ratio of the measurements $M = \log_2 a - \log_2 b$ against the log of their geometrical mean $A = 0.5 \cdot (\log_2 a + \log_2 b)$. Here a and b are density and density ratio measurements for the same gene (segment) from two different replicates. These plots were made for full-length CDSes and for segment trees.

MA plots for the full length genes (~~Figure~~Fig SI-2) were used to manually set the total read count thresholds for reliable density estimates. The chosen thresholds of 128 reads are identical to the ones used in Ingolia *et al.* [6] for defining reliably measured genes.

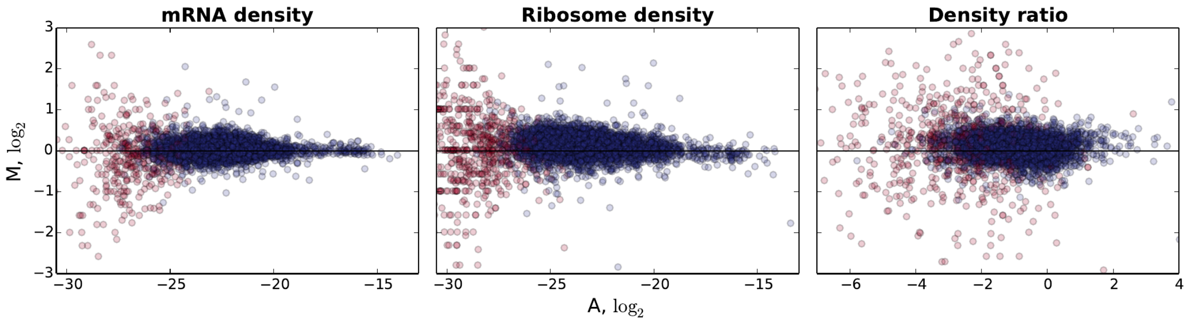

Figure SI-2. MA-plot of the full-CDS density estimates. Measurement variance for ribosome and mRNA density estimates is higher for genes with low density. Unreliable density estimates ($<$ 128 total RNA-seq reads or $<$ 128 ribo-seq reads; colored red) show increased measurement variance.

SI-3 Segment tree construction

When constructing segment trees, cut points p are chosen such that the combined number of RNA- and ribo-seq reads across replicates is divided equally between the left and the right segments. This is achieved by simultaneously minimizing for the available replicates the sum of absolute per-replicate differences in the combined numbers of RNA- and ribo-seq reads between the left and the right segments.

When recursively splitting segments, cuts where both segments pass the minimum length criterion are preferred to cuts that minimize the read count imbalance. If multiple cut points with the same imbalance are available, the leftmost one is chosen. Measurements from segments, in which one or more density estimates are based on read counts containing $\geq 20\%$ ambiguous or overlapping reads with other CDSes, are discarded, but tree construction is allowed to continue.

SI-4 Density-dependent bias correction

MA-plots in ~~Figure~~Fig SI-3 (i) suggest presence of a systematic density-dependent bias in density and ratio measurements. To remove the bias, density measurements were normalized by first estimating the local bias using LOWESS regression (red line in MA-plots) and then (a) subtracting it from M and (b) subtracting a half of it from A. Although this bias is negligible for mRNA and density measurements, it may get amplified when the ratio of the two biased measurements is computed. This effect can already be seen from the density ratio MA-plots, where the bias only becomes more pronounced after density

normalization. The remaining bias in the density ratio estimates is removed be applying the normalization procedure to the ratio estimates (~~Figure~~Fig SI-4).

We note that although this bias correction procedure does not allow for removing bias from the original measurements, it provides bias-corrected versions of quantities M and A, which are sufficient to determine parameters of the log-normal distributions describing the segment tree measurements. Quantity A directly gives the scale parameter μ of the distribution for the corresponding segment, and M is essentially the i.r.e. which is used to determine segment length group shape parameters.

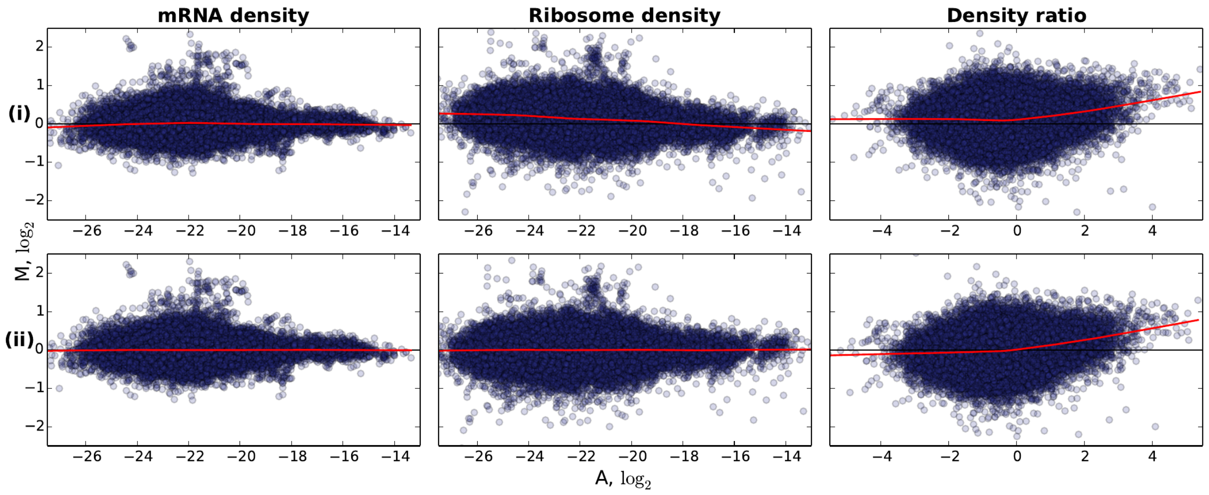

Figure SI-3. MA-plots of the segment tree density and ratio measurements (i) before bias correction show a density-dependent systematic bias, which (ii) after removal of mRNA and ribosome density bias is no longer present in density estimates, but is amplified for the ratio estimates. An identical procedure is applied to correct this amplified bias (see ~~Figure~~Fig SI-4). The locally estimated mean $\bar{M}$ (red line) was obtained using LOWESS [7] using 33% of the data.

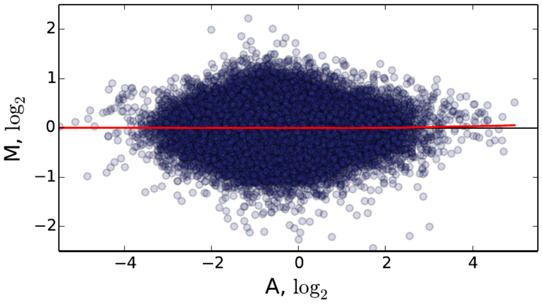

Figure SI-4. MA-plot of the segment tree density ratio estimates after normalization shows that no significant bias is present at the extreme density ratio values.

SI-5 Estimating shape parameters from i.r.e.

To estimate shape parameters σ_j of the log-normal distributions $\ln\mathcal{N}(\mu_j, \sigma_j)$ describing density ratio measurements, segments were divided into 10 groups based on their length. Because each segment in a group follows a different distribution, with its own parameter μ_j, the per-group shape parameter cannot be estimated directly from the density ratio measurements. Instead, for each group k we estimate it via the shape parameter $\sigma_k^{\text{i.r.e.}}$ of the i.r.e. for measurements in this group. If $X, Y \sim \ln\mathcal{N}(\mu_j, \sigma_j)$ are random variables representing two independent replicated measurements of the density ratios, then the shape parameters of the i.r.e. and the density ratio distributions are related as

$$\left(\sigma_k^{\text{i.r.e.}}\right)^2 = \text{Var}(X) + \text{Var}(Y) = 2 \cdot (\sigma_k^{\text{group}})^2 \tag{1}$$

Using this equation the group shape parameter is calculated as $\sigma_k^{\text{group}} = \frac{1}{\sqrt{2}}\sigma_k^{\text{i.r.e.}}$ and used in place of σ_j for all segments in the group.

SI-5.1 Variance structure in segments with high and low initiation rates

It is possible that segments originating from genes with high initiation rates have a different variance structure (e.g. are more reliable) than genes with low initiation rates. If present, this kind of relationship would be missed by the proposed segment grouping strategy and render it problematic. To confirm that gene initiation rates do not significantly alter variance structure of their corresponding segments we plotted inferred gene initiation rates from several existing datasets [8–10] against the segments inter-replicate errors (i.e. M from the MA-plots) used for estimating the log-normal distribution shape parameters σ_k^{group} as described before. ~~Figures~~Figs SI-5 to SI-7 show that no strong relationship between the initiation rates and inter-replicate errors is present. Only for initiation rates obtained from Shah *et al.* [10] (~~Figure~~Fig SI-7) there appears to be a weak tendency of increasing i.r.e. for lower initiation rates. We believe that absence of strong dependencies between initiation rates and i.r.e. justifies our segment grouping approach and use it to derive log-normal distribution shape parameters as described above.

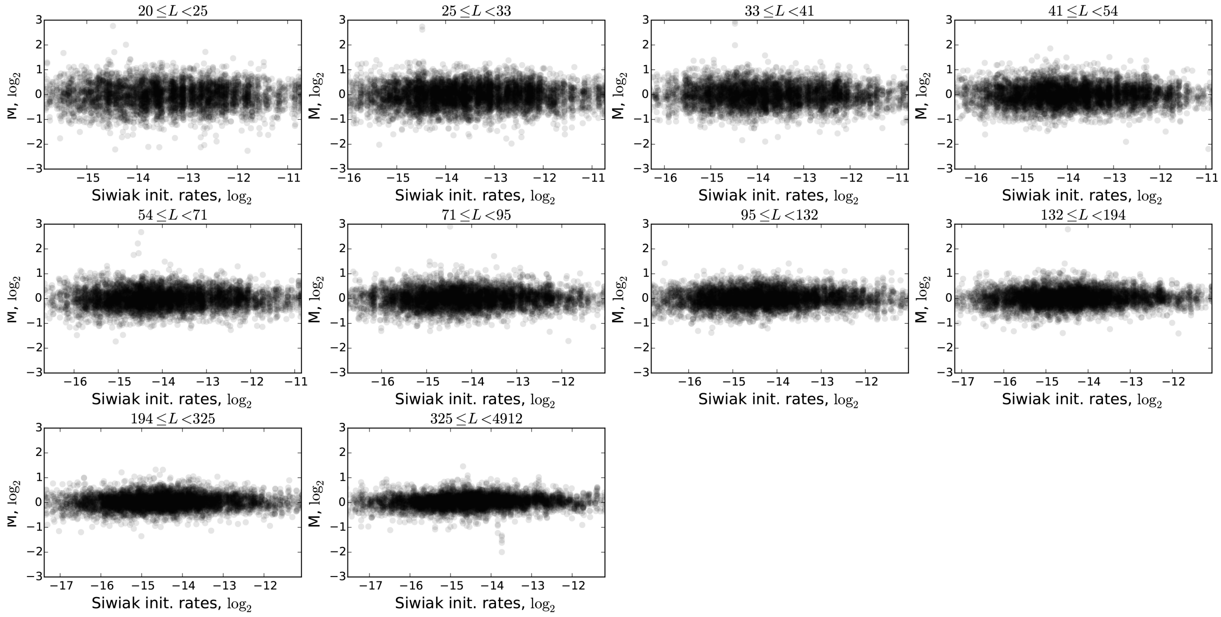

Figure SI-5. Density ratio i.r.e. (M from the MA-plot in ~~Figure~~Fig SI-4) plotted against initiation rates obtained from Siwiak and Zielenkiewicz [8] for each of the 10 segment length groups used in the main text. No relationship between i.r.e. and initiation rates can be observed.

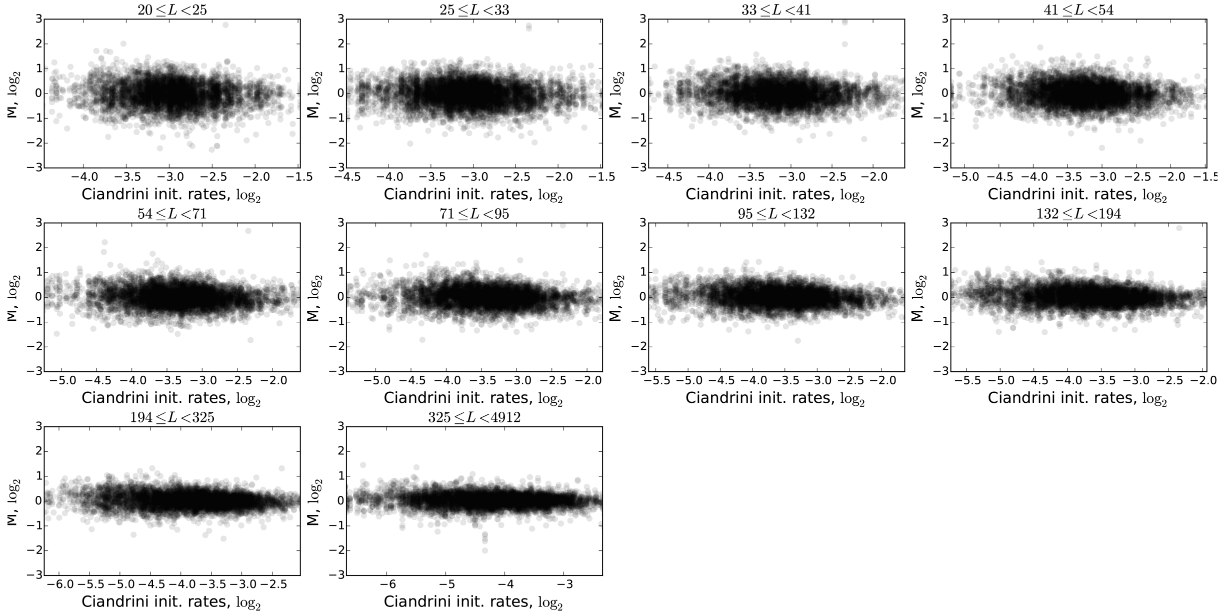

Figure SI-6. Density ratio i.r.e. M plotted against initiation rates obtained from Ciandrini *et al.* [9] for each of the 10 segment length groups used in the main text. No relationship between i.r.e. and initiation rates can be observed.

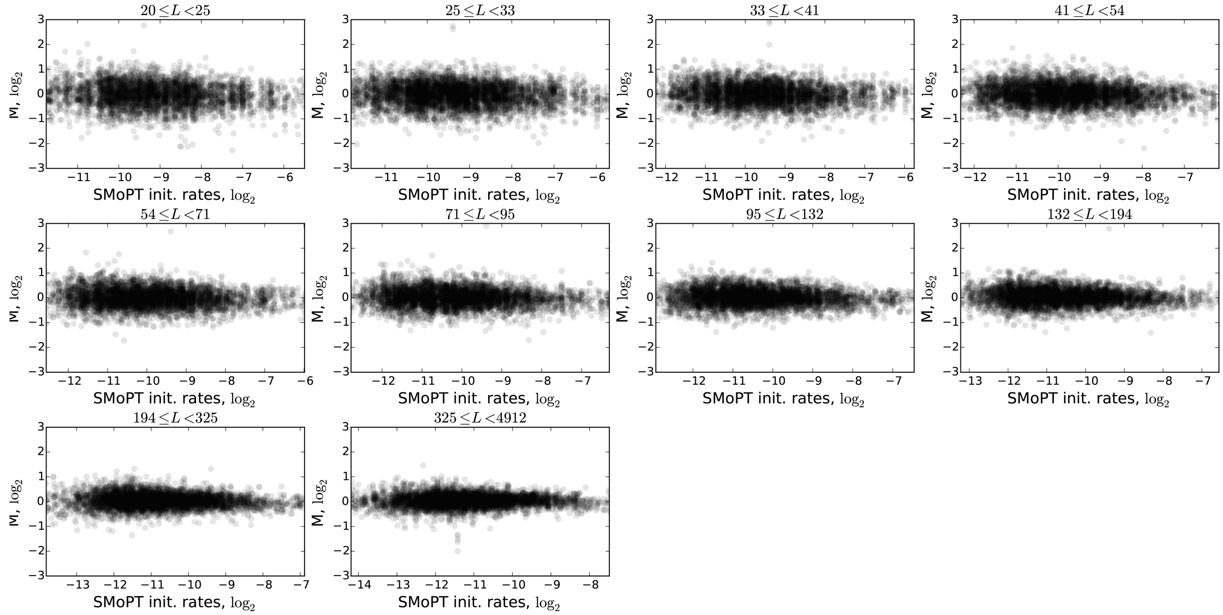

Figure SI-7. Density ratio i.r.e. M plotted against initiation rates obtained from Shah *et al.* [10] for each of the 10 segment length groups used in the main text. Only a weak relationship (the scatterplot has a droplet-like shape if viewed horizontally; clearly seen for group $194 \leq L < 325$) between i.r.e. and initiation rates can be observed.

SI-6 Ribosome occupancy profiles

The mRNA and ribosome occupancy profiles were obtained by assigning reads to coding sequences as in the case of segment tree construction. The nucleotide occupancy counts were then normalized by dividing them by N_R or N_M (the total number of ribo- and RNA-seq reads mapped to CDSes) depending on the profile; and the normalized counts were coarse-grained into codon-resolution count profiles by summing counts over nucleotide positions of the corresponding codons.

To obtain per-transcript ribosome occupancy profiles, the ribosome count profiles were further normalized in two different ways: either by dividing the per-position counts of the ribosome profiles by the average count of the corresponding mRNA profile (referred to as mean normalization); or by dividing per-position counts of the ribosome profiles by the respective (same CDS and same position in the CDS) counts of the mRNA profiles (referred to as profile normalization). The latter normalization method is conceptually similar to the way in which density ratios in segment trees are calculated, but is applied at single codon resolution.

SI-6.1 High variance of RP measurements at single codon resolution complicates inference of translation kinetics

Prior to choosing for a "multi-scale" segment tree approach to interpreting the RP data, we evaluated its *quantitative* reproducibility at single-codon resolution by comparing ribosome occupancy profiles between replicates. To this end we obtained occupancy profiles using either profile or mean normalizations (PN and MN respectively). Separate ribosome occupancy profiles were obtained for the available biological replicates. For each reliably measured gene (as defined in Section SI-2) Pearson correlation between profiles obtained from the two replicates were calculated. Profile positions, for which it was impossible to obtain a profile (e.g. due to zero mRNA profile counts) in at least one of the replicates, were left out of the analysis.

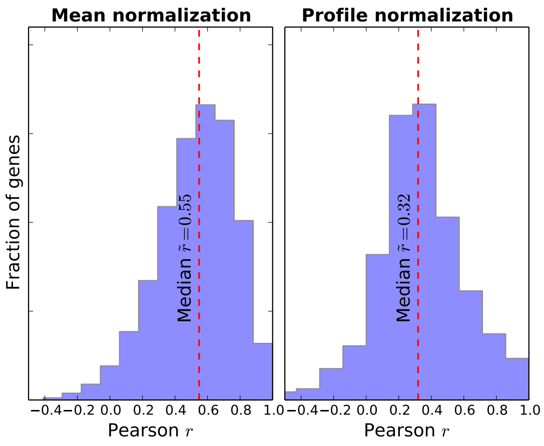

Figure SI-8. Histograms of Pearson correlation coefficients for ribosome occupancy profiles obtained from two biological replicates demonstrate limited reproducibility of the profiles in a majority of the reliably measured genes irrespective of the used normalization method.

We found that profile correlations demonstrate limited agreement of the ribosome occupancy profiles (median correlation coefficient $\tilde{r} = 0.55$; ~~Figure~~Fig SI-8, left). This conclusion does not change even when more stringent read filtering is used (~~Figure~~Fig SI-9). Since PN profiles can be viewed as an extreme case of a segment tree, where segments do not overlap and are one codon in length, they were computed

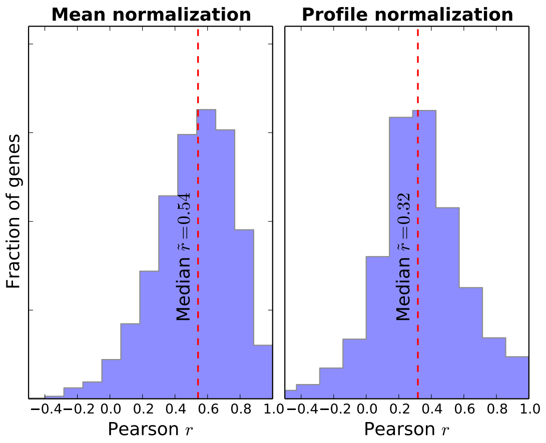

Figure SI-9. Histograms of Pearson correlation coefficients for ribosome occupancy profiles obtained from two biological replicates. Only uniquely mapping reads with at most 1 mismatch were used to construct the profiles (see Table SI-2 for statistics on read mapping). Nonetheless, the resulting correlation coefficients did not improve compared to the case of using less stringent read filtering (~~Figure~~Fig SI-8).

as a reference for the segment tree interpretation. We found that PN scheme performed worse than MN (median correlation coefficient of $\tilde{r} = 0.32$; ~~Figure~~Fig SI-8, right), presumably because it introduced additional noise into the profiles through division by poorly estimated counts.

In the original publication Ingolia *et al.* [6] showed that RP data has good reproducibility when analyzed at whole-gene scale. Given the low Pearson correlation coefficients obtained for occupancy profiles evaluated at single codon resolution, we concluded that the RP data interpreted at single codon resolution would not allow for quantitative inference of translation kinetics; and devised a segmentation approach that estimates (local) average ribosome occupancy of a gene at multiple scales.

SI-7 Objective function derivation

In order to derive the objective function we assume that the density ratios obtained from the RP data follow the log-normal distribution. Further we assume that measurements for different genes and segments are independent from each other. We then seek to quantify how likely it is that a certain *simulated* segment density matches the measured one by plugging it into the log-normal probability density function (PDF) of that segment. The objective function can then be written as a product of PDFs of individual segments:

$$F\left(n|T\right) = \prod_{g} \prod_{j \in J^g} f_x\left(N_j^g; \mu_j^g, \sigma_j^g\right),$$

where $f_x\left(x; \mu, \sigma\right) = \frac{1}{x\sigma\sqrt{2\pi}} e^{-\frac{(\ln x - \mu)^2}{2\sigma^2}}$ is the log-normal PDF and other variables hold the following meaning:

- g denotes the gene.
- J^g denotes the set of segments of gene g.
- j denotes the segment within this gene.
- μ_j^g and σ_j^g are the scale and shape parameters of the log-normal distribution describing density ratios of segment j from gene g.

- T denotes the set of μ_j^g and σ_j^g for all genes and segments.
- n denotes the simulated ribosome occupancy for all genes at single codon resolution.
- N_j^g denotes the average simulated ribosome occupancy for segment j of gene g, which is matched against the estimated density ratio of the same segment.

The comparison of simulated average ribosome occupancy and the estimated density ratios is complicated by the fact that these are measured on different scales. We therefore need to rescale the simulated occupancy and the measured data to the same scale. We do this by scaling the ratio density distributions by a factor $\frac{1}{C}$, which for the moment we assume to be known. This is equivalent to transforming the PDF of the measured data into the PDF of the simulated occupancy and using the latter to evaluate simulation results. To derive the transformed PDF $f_y(y;\mu,\sigma)$ we apply Theorem 5.11 from [11].

Let x be the random variable with PDF $f_x(x;\mu,\sigma)$ representing the data distribution and let $y = \frac{1}{C}x$ be the rescaled version of this random variable that is on the simulation scale. The goal is then to determine $f_y(y;\mu,\sigma)$, i.e. the PDF of the rescaled variable. According to Theorem 5.11 f_y can be written as

$$f_y(y;\mu,\sigma) = \frac{1}{\frac{1}{C}} f_x(\frac{1}{\frac{1}{C}}y) = Cf_x(Cy) = \frac{C}{Cy\sigma\sqrt{2\pi}}e^{-\frac{(\ln(Cy)-\mu)^2}{2\sigma^2}} = \frac{1}{y\sigma\sqrt{2\pi}}e^{-\frac{(\ln y+\ln C-\mu)^2}{2\sigma^2}} =$$
$$= \frac{1}{y\sigma\sqrt{2\pi}}e^{-\frac{(\ln y-(\mu-\ln C))^2}{2\sigma^2}} = f_x(y;\mu-\ln C,\sigma)$$

We can now go back to the objective function $F(n|T)$ and replace f_x with f_y (denoted as f_C in the main text):

$$F(C,n|T) = \prod_g \prod_{j\in J^g} f_x\left(N_j^g;\mu_j^g-\ln C,\sigma_j^g\right) = \prod_g \prod_{j\in J^g} \frac{1}{N_j^g\sigma_j^g\sqrt{2\pi}} e^{-\frac{\left(\ln N_j^g-\mu_j^g+\ln C\right)^2}{2\left(\sigma_j^g\right)^2}}$$

To avoid working with multiplications, we will instead consider the logarithm of F:

$$\ln F(C,n|T) = \sum_g \sum_{j\in J^g} \left[-\frac{\left(\ln N_j^g-\mu_j^g+\ln C\right)^2}{2\left(\sigma_j^g\right)^2} + \ln\left(\frac{1}{N_j^g\sigma_j^g\sqrt{2\pi}}\right)\right] =$$
$$= \sum_g \sum_{j\in J^g} \left[-\frac{1}{2\left(\sigma_j^g\right)^2}\left(\ln N_j^g-\mu_j^g+\ln C\right)^2 - \ln\left(N_j^g\sigma_j^g\sqrt{2\pi}\right)\right] =$$
$$= \sum_g \sum_{j\in J^g} \left[-\frac{1}{2\left(\sigma_j^g\right)^2}\left(\ln N_j^g-\mu_j^g+\ln C\right)^2 - \ln N_j^g - \ln\left(\sigma_j^g\sqrt{2\pi}\right)\right]$$

If we now drop the constants from $\ln F(C,n|T)$, we get the final objective function:

$$\psi(C,n|T) = \sum_g \sum_{j\in J^g} \left[-\frac{1}{2\left(\sigma_j^g\right)^2}\left(\ln N_j^g-\mu_j^g+\ln C\right)^2 - \ln N_j^g\right].$$

We now address an earlier assumption that the scaling factor C is known. To this end we find a C that maximizes the objective ψ when all other variables are given (i.e. when the simulated occupancy n and the parameters of the log-normal distributions are available). To this end we take the derivative of ψ with respect to $\ln C$ and equate it to zero:

$$\frac{\partial \psi}{\partial \ln C} = \sum_g \sum_{j \in J^g} \left[-\frac{2}{2\left(\sigma_j^g\right)^2} \left(\ln N_j^g - \mu_j^g + \ln C\right) \right] = \sum_g \sum_{j \in J^g} \left[-\frac{1}{\left(\sigma_j^g\right)^2} \left(\ln N_j^g - \mu_j^g + \ln C\right) \right] = 0$$

$$\begin{aligned}
\sum_g \sum_{j \in J^g} \left[-\frac{1}{\left(\sigma_j^g\right)^2} \left(\ln N_j^g - \mu_j^g + \ln C\right) \right] &= 0 \\
\sum_g \sum_{j \in J^g} \frac{1}{\left(\sigma_j^g\right)^2} \left(\ln N_j^g - \mu_j^g\right) + \sum_g \sum_{j \in J^g} \frac{1}{\left(\sigma_j^g\right)^2} \ln C &= 0 \\
\sum_g \sum_{j \in J^g} \frac{1}{\left(\sigma_j^g\right)^2} \left(\ln N_j^g - \mu_j^g\right) = -\sum_g \sum_{j \in J^g} \frac{1}{\left(\sigma_j^g\right)^2} &\ln C \\
-\sum_g \sum_{j \in J^g} \frac{1}{\left(\sigma_j^g\right)^2} \left(\ln N_j^g - \mu_j^g\right) = \sum_g \sum_{j \in J^g} \frac{1}{\left(\sigma_j^g\right)^2} &\ln C \\
\sum_g \sum_{j \in J^g} \frac{1}{\left(\sigma_j^g\right)^2} \left(\mu_j^g - \ln N_j^g\right) = \sum_g \sum_{j \in J^g} \frac{1}{\left(\sigma_j^g\right)^2} &\ln C \\
\frac{\sum_g \sum_{j \in J^g} \frac{1}{\left(\sigma_j^g\right)^2} \left(\mu_j^g - \ln N_j^g\right)}{\sum_g \sum_{j \in J^g} \frac{1}{\left(\sigma_j^g\right)^2}} &= \ln C
\end{aligned}$$

The end result

$$\ln C = \frac{\sum_g \sum_{j \in J^g} \frac{1}{\left(\sigma_j^g\right)^2} \left(\mu_j^g - \ln N_j^g\right)}{\sum_g \sum_{j \in J^g} \frac{1}{\left(\sigma_j^g\right)^2}}$$

also allows an appealing interpretation as the weighted sum of scale differences and matches the intuition about the scaling factor.

SI-8 Initiation rate approximation

We propose a method for approximating initiation rates that is based on the observation of Ciandrini *et al.* [9] that the average number of ribosomes bound to a segment (i.e. the segments average ribosome occupancy) as a function of the initiation rate, $A(k_0)$, reaches saturated state either smoothly or abruptly (~~Figures~~Figs SI-10 (i) and SI-11). This limited set of steady-state behaviors allows for efficiently approximating $A(k_0)$. For each segment j we approximate the *shape* of this function as

$$\begin{aligned}
f_j(k_0) &= \begin{cases} f_j^-(k_0), & k_0 \leq e_j \\ f_j^+(k_0), & k_0 > e_j \end{cases} \\
f_j^-(k_0) &\equiv a_j k_0/(b_j + k_0) \quad , \\
f_j^+(k_0) &\equiv \; c_j k_0 + d_j
\end{aligned}$$

where $a_j, b_j, c_j \geq 0$, and d_j are parameters that need to be determined. Here $f_j^-(k_0)$ and $f_j^+(k_0)$ are used to approximate the unsaturated and saturated parts of $A_j(k_0)$ respectively. Approximation parameters are iteratively updated inside a bracket search (~~Figure~~Fig SI-10 (ii)). The approximation of $A_j(k_0)$ is then used to approximate gene initiation rates as discussed below.

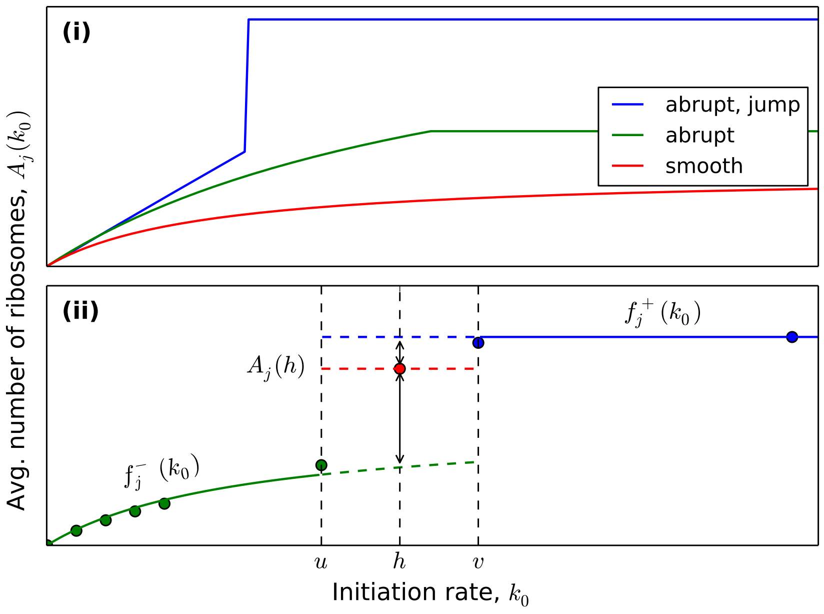

Figure SI-10. $A_j(k_0)$, the average number of ribosomes attached to segment j at steady-state increases as a function of initiation rate k_0 and reaches saturated state (i) smoothly, abruptly or abruptly with a "jump"; this observation can be used to efficiently approximate the shape of $A_j(k_0)$. At each step of the bracket search (ii) the decision whether the gene is saturated at initiation rate h, the midpoint of bracket $[u, v]$, is made based on the squared distance from $A_j(h)$ (red dashed line) to $f_j^-(h)$ (green lines) and $f_j^+(h)$ (blue lines) - the approximation functions fitted into model simulation results (circles); and the approximations are refit in accordance to the decision made.

In order to approximate initiation rates, we assume that codon elongation rates are given and a "proposed" scaling factor $\tilde{C}$, an estimate of the unknown true scaling factor C, is available. Gene initiation rates k_0 are then chosen to maximize the objective $\psi(C, n|T)$ for $\tilde{C}$ and the approximations $f_j(k_0)$ obtained earlier. In practice the objective evaluated for $f_j(k_0)$ is a unimodal function of the initiation rate (see ~~Figure~~Fig SI-12) and ternary search is used to efficiently find the k_0 that maximizes it, i.e. the sought initiation rate approximation.

To determine approximation parameter values for segment j, the model is simulated for E low and high initiation rates and f_j^- and f_j^+ are first fit onto points $(k_0, A_j(k_0))$ recorded for low and high initiation rates respectively by minimizing the summed squared error. $E = 5$ was used as it gives robust estimates in practice and the low and high initiation rates are equally spaced in $(0, \min_i k_i/2]$ and $[\max_i k_i, 1]$ respectively. Bracket search is then used to find the switch point e_j.
Starting from bracket $[u, v] = [0, 1]$, a midpoint $h = (u + v)/2$ is chosen and $A_j(h)$ is found by simulation. $A_j(h)$ is compared to $f_j^-(h)$ and $f_j^+(h)$ to determine whether at initiation rate h the mRNA is already in saturated state, and the bracket is updated as

$$\begin{aligned} u \leftarrow h, &\quad \text{if } \left(f_j^-(h) - A_j(h)\right)^2 < \left(f_j^+(h) - A_j(h)\right)^2 \\ v \leftarrow h, &\quad \text{otherwise.} \end{aligned}$$

Parameters a_j, b_j or c_j, d_j are then refit with the new point $(h, A_j(h))$ depending on whether the left or the right edge of the bracket was changed. The process continues until the bracket length becomes smaller

than 10^{-6} and the switch point is then calculated as $e_j = (u + v)/2$.
Approximations $f_j(k_0)$ are obtained for each segment j and are recomputed each time elongation rates change.

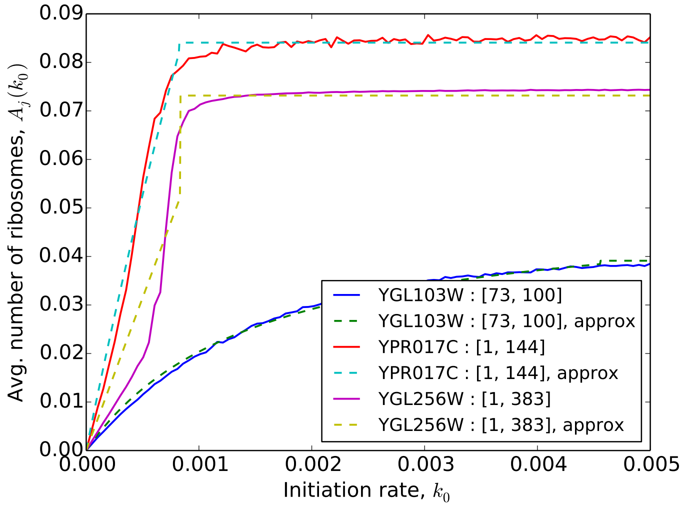

Figure SI-11. Examples of the three types of behavior of the average number of ribosomes $A_j(k_0)$ as a function the initiation rate k_0 (solid lines) and their approximations (dashed lines): smooth - YGL103W (blue, green); abrupt - YPR017C (red, cyan); abrupt jump - YGL256W (magenta, yellow). tAI-based elongation rates were used in simulations.

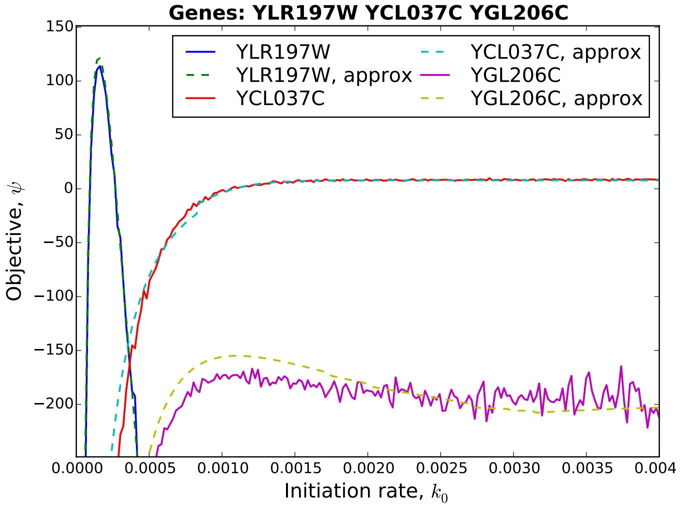

Figure SI-12. Objective function value for genes YLR197W, YCL037C and YGL206C plotted as a function of the initiation rate for proposed scale $\tilde{C} \approx 181.956$ (see next section). The objective calculated using the approximation of the average number of ribosomes (dashed lines) and the objective calculated based on the simulation output (solid lines) demonstrate near-identical behavior. tAI-based elongation rates were used in simulations.

SI-9 The proposed scaling factor $\tilde{C}$

The proposed scaling factor $\tilde{C}$ and the scaling factor C used in the objective function ψ are both responsible for matching scales between simulated ribosome occupancy and the measured data. However, unlike the freely changing parameter C, the proposed scale $\tilde{C}$ is fixed and defines which portion of the difference between the simulated and measured densities (N_j^g and μ_j^g respectively) can be attributed to the scale mismatch, and which portion must be explained the TASEP model. Choosing $\tilde{C}$ to be a good estimate of the unknown true scaling factor is required to ensure that the fitted translation rates have biologically meaningful values.

The true scaling factor is impossible to measure exactly, but it is possible to estimate it. It is determined by the amount of DNA available for sequencing, which in turn depends on the number of actively translating ribosomes (for ribo-seq), the total size of the coding transcriptome (for RNA-seq) and the efficiencies of individual steps of the experimental protocol. To estimate this factor we assume that individual steps of the ribosome profiling protocol are either highly efficient (i.e. only a moderate portion of the genetic material and ribosomes are lost during their execution), or that they are equally inefficient for the ribo-seq and RNA-seq measurements, and use the procedures from Siwiak and Zielenkiewicz [8] to estimate the number of actively translating ribosomes P_{active} and the size of the coding transcriptome Q.

The total number of ribosomes $P_{\text{total}} = 2 \times 10^5$ and the fraction of ribosomes involved in active translation $\rho = 0.85$ [12] were used to calculate $P_{\text{active}} = \rho \cdot P_{\text{total}} = 1.7 \times 10^5$. The size of the coding transcriptome was computed as

$$Q = 3 \cdot \sum_g S^g K_g,$$

where S^g and K_g are respectively the length in codons of CDSes and the absolute number of transcripts of gene g. Quantities K_g were calculated as the relative mRNA abundance $m_g = \frac{M_{[1,S^g]}}{N_M}$ scaled by the total number of mRNA molecules per cell $E = 36{,}139$ [13], yielding $Q = 3E \cdot \sum_g S^g m_g \approx 3.09 \times 10^7$.

Using these quantities the proposed scaling factor can be estimates as

$$\tilde{C} = \frac{Q}{P_{\text{active}}} \approx 181.956.$$

The described procedure was used to set the proposed scale $\tilde{C}$ for all analyzed datasets individually.

SI-10 CMA parameter settings

CMA search space was constrained to $[-12, 12]$. This way, when sigmoid-transformed prior to TASEP simulations, the rates would occupy the interval $(0, 1)$ almost entirely. When fitting elongation rates, rates consistent with the tRNA pool adaptation hypothesis were used as a starting point and their standard deviation (SD) was used to set CMA parameter $\sigma_0 \approx 0.94$. CMA was run with the default population size $\mu = 16$. To control the runtime of the algorithm, it was stopped if the search ran for at least 300 generations and the overall best solution did not improve over the last 50 generations.

SI-11 Comparison to other models

Zhang's model [14] was designed to predict the relative local speed of translation at a given position from codon elongation rates around it. Codon elongation times t_i (inverse of elongation rates) consistent with the tRNA pool adaptation hypothesis were used to parameterize it as in Wohlgemuth *et al.* [15].

To obtain per-gene translation time profiles from the Zhang model, individual codon translation times t_i were smoothed with a moving average window of 19 codons as in the original publication. For model evaluation these profiles were treated as codon occupancy probabilities output by other models.

SMoPT (Stochastic Model of Protein Translation [10]) is a full-cell model developed and parameterized for yeast using the RP data [6]. It describes the movement of ribosomes on mRNA transcripts with a TASEP-like process while also taking tRNA and ribosome concentration into account.

To obtain ribosome occupancy profiles, the model was simulated with default settings for the maximum allowed time (2.4×10^6 seconds; ~~Figure~~Fig SI-13) and snapshots of the state of the model with exact locations of ribosomes on all transcripts were taken every second. These snapshots were processed into ribosome occupancy estimates by recording how often a ribosome was seen at a particular location and dividing this number by the total simulation time. Observations for different transcripts of the same gene were combined into a single occupancy profile normalized by the number of transcripts. Since SMoPT implicitly assumes that termination is instantaneous, codon occupancies for stop codons were set to zero.

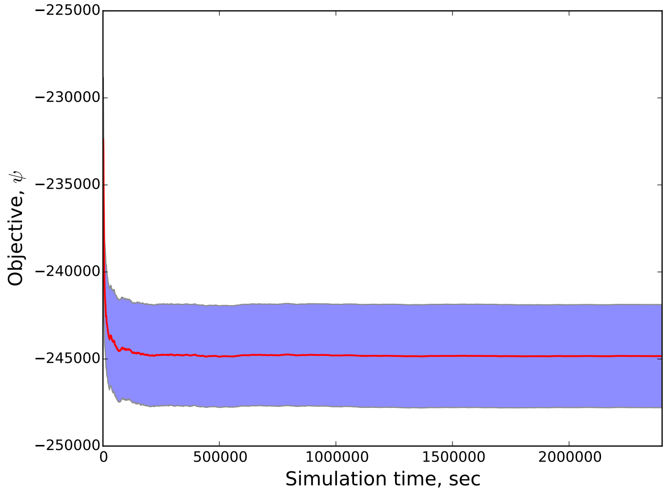

Figure SI-13. CV objective ψ for the SMoPT simulation output (red line) and its standard deviation (blue area) plotted as the function of the simulation time demonstrates that reliable density estimates were determined by the end of the simulation period.

SI-12 Translation rate reproducibility analysis

In order to determine how robust translation rates fitted on the McManus *et al.* [1] dataset are, we set out to repeat model fitting on an independent dataset. The RP data for yeast *Saccharomyces cerevisiae* strain BY4741 [6] was used for this purpose. It is available as a read mapping against the reference genome sequence of *S. cerevisiae* strain S288C taken from SGD on June 22, 2008 (release R58). These data were re-mapped to release R64 (January 14, 2014) by sequentially updating the alignment coordinates according to the sequence changes file available from SGD. The updated alignments were filtered as in in the original publication. We intentionally did not use a stringent cutoff and kept alignments with up to 2 mismatches, as we expect that a fraction of the mismatches originates from the use of the S288C reference genome for reads of a different strain. After discarding footprint lengths for which location of the A-site could not be reliably determined, reads of lengths $22 \leq l \leq 32$ and $27 \leq l \leq 32$ for mRNA- and ribo-seq reads respectively were used in the analysis. A-site determination (~~Figure~~Fig SI-14 and Table SI-3) and assignment of reads to CDSes (see Table SI-4 for statistics) were performed as before.

MA-plots for the full length genes (~~Figure~~Fig SI-15) were similarly used to set read count thresholds for reliable density estimates to 128 and 64 mRNA- and ribo-seq reads respectively. Segment trees constructed with these thresholds were then bias-corrected (~~Figures~~Figs SI-16 and SI-17) and ratio measurement errors (Table SI-5) were determined as before.

The set of 2,949 genes common between the McManus *et al.* [1] and Ingolia *et al.* [6] datasets with 13,443 Ingolia and 51,223 McManus segments was then used to independently fit two TASEP^{elong} models on the two datasets inside a common 5-fold CV loop.

Table SI-3. P-site offsets for various footprint lengths for the Ingolia *et al.* [6] dataset determined individually for each replicate based on ~~Figure~~Fig SI-14.

Length	Replicate 1		Replicate 2		Final offset
	Start	Stop	Start	Stop	
27	12	12	13	12	12
28	12	12	12	12	12
29	13	12	13	12	13
30	13	13	13	13	13
31	13	13	13	13	13
32	12	12	12	NA	12

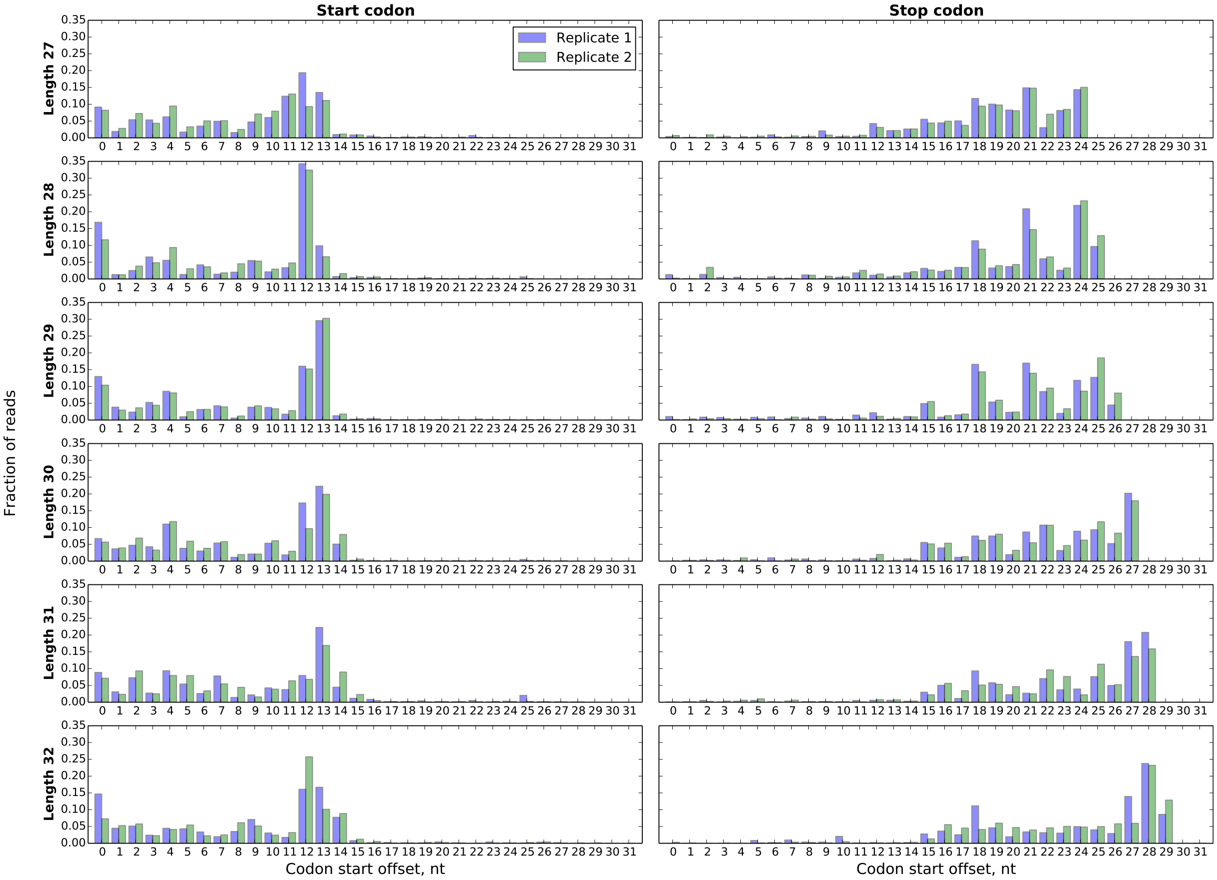

Figure SI-14. Histograms of the locations of the start and stop codons within ribosomal footprints of various lengths for the Ingolia *et al.* [6] dataset. Offsets fixed for every footprint length are given in Table SI-3.

Table SI-4. Ingolia dataset [6] read alignment statistics for the *S. cerevisiae* genome release used in the original publication (R58) and the genome release used in this study (R64), and varying alignment strictness. The switch from R58 to R64 gives an increase in the number of reads with up to 2 mismatches.

Type	Release	Name	Max subst.	Min length	Max length	Unique	Replicate 1		Replicate 2	
							mRNA	Ribosome	mRNA	Ribosome
Ref.	R58	Raw	2	20	35	No	2,219,009	1,868,620	4,895,436	3,424,182
	R64	Raw	2	20	35	No	2,226,949	1,870,581	4,898,568	3,439,420
Ref.	R64	Filtered	2	22/27	32	No	1,924,200	1,175,283	4,583,654	1,699,871
		Strict	1	22/27	32	No	1,825,219	1,109,120	3,643,440	1,281,387
		Unique	1	22/27	32	Yes	1,618,952	926,029	3,216,498	1,062,049
CDS	R64	Filtered	2	22/27	32	No	1,365,482	1,128,724	3,451,402	1,649,033
		Strict	1	22/27	32	No	1,297,286	1,069,840	2,729,730	1,245,578
		Unique	1	22/27	32	Yes	1,125,445	897,963	2,369,133	1,034,200

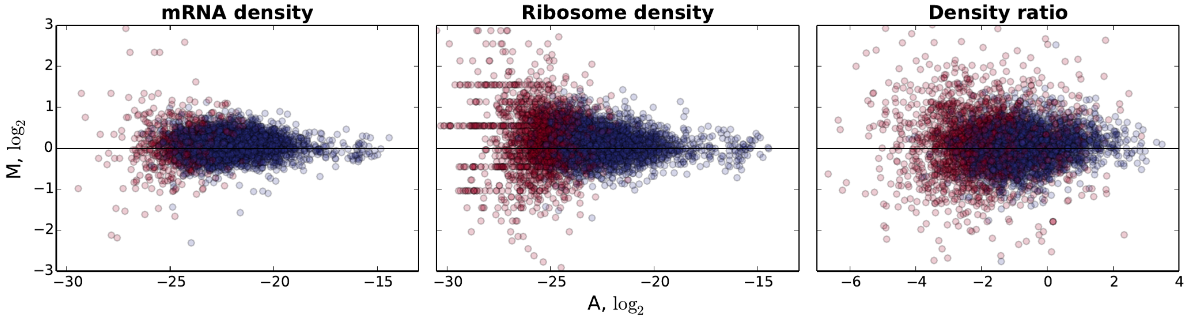

Figure SI-15. MA-plot of the full-CDS density estimates computed for the Ingolia *et al.* [6] dataset. Unreliable density estimates (< 128 total RNA-seq reads or < 64 ribo-seq reads; colored red) show increased measurement variance.

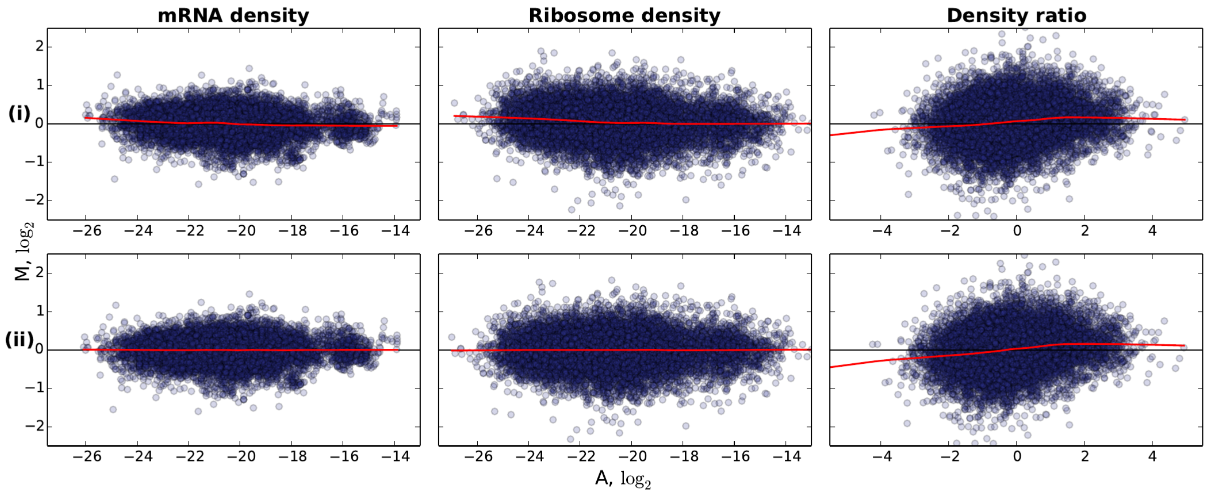

Figure SI-16. MA-plots of the segment tree density and ratio measurements for the Ingolia *et al.* [6] dataset (i) before bias correction show a density-dependent systematic bias, which (ii) after removal of mRNA and ribosome density bias is no longer present in density estimates. An identical procedure is applied to correct density-dependent amplified in ratio measurements (see ~~Figure~~Fig SI-17).

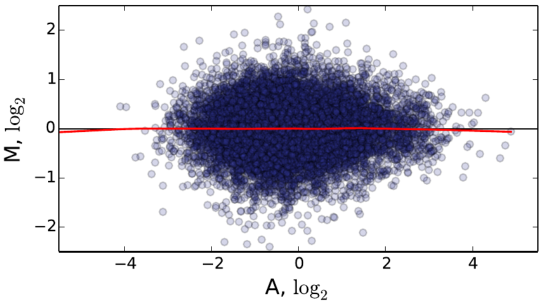

Figure SI-17. MA-plot of the segment tree density ratio estimates of the Ingolia *et al.* [6] dataset after normalization shows that no significant bias is present at the extreme density ratio values.

Table SI-5. Shape parameters of the Ingolia *et al.* [6] dataset density ratio distributions for segments grouped by length. Values of σ determined for this dataset are considerably higher than the values obtained for McManus data.

#	Left	Right	Group size	Shape parameter σ, $\log_2$
1	20	31	1304	0.320130113060900
2	31	47	1400	0.292439449293707
3	47	67	1359	0.278283672264551
4	67	94	1366	0.261941203773427
5	94	130	1399	0.245270164406985
6	130	176	1359	0.239063834104316
7	176	246	1353	0.225808138327335
8	246	354	1375	0.214356415855909
9	354	560	1374	0.206160115988558
10	560	4912	1367	0.199898527308120

SI-12.1 Model fitting is robust against experimental biases

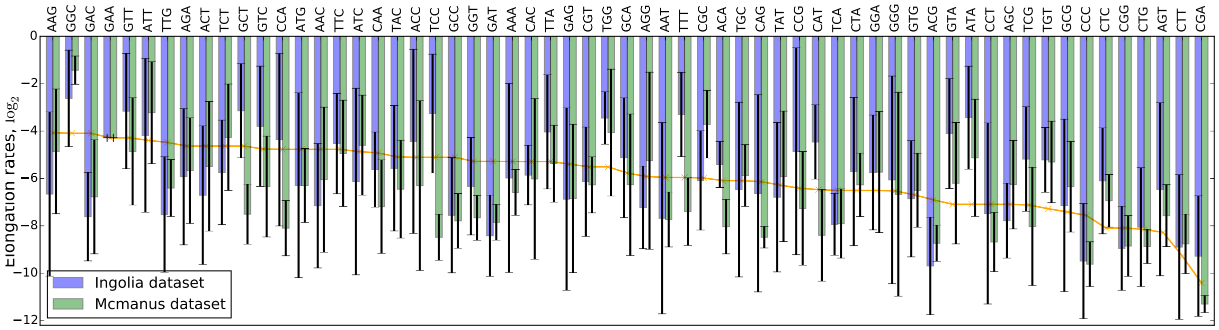

Figure SI-18. Mean and SD of the codon elongation rates fitted in different CV folds of McManus and Ingolia datasets, compared to the tAI-based rates (orange line). Many codons show agreement between the elongation rates fitted on the Ingolia dataset (blue) and the elongation rates fitted on the McManus dataset (green).

Translation rates obtained from the two datasets (~~Figure~~Fig SI-18) and initiation rates show qualitatively similar behaviour (~~Figure~~Fig SI-19) despite of significant differences in protocols, strains, sequencing depth and computational processing between the two datasets. Ingolia *et al.* [6] describe the first application of the ribosome profiling method. They used a RP protocol based on poly-A tailing of ribosomal footprints, which was later substituted by ligation of an adapter sequence to the 3′ end (e.g. McManus *et al.* [1]). The latter is a standard procedure in small RNA sequencing, as it simplifies the experimental protocol and subsequent short read mapping. This difference in protocols results in substantially different biases due to sequence preferences of poly-A polymerase and RNA ligase [16], which could explain the discrepancies between the elongation rates fitted on the two datasets. Relatively low sequencing depth of the Ingolia *et al.* dataset is also a likely factor contributing to higher SDs of the fitted elongation rates and moderate reproducibility. Nevertheless, the general agreement between the two sets of translation rates shows that our approach is robust against experimental biases.

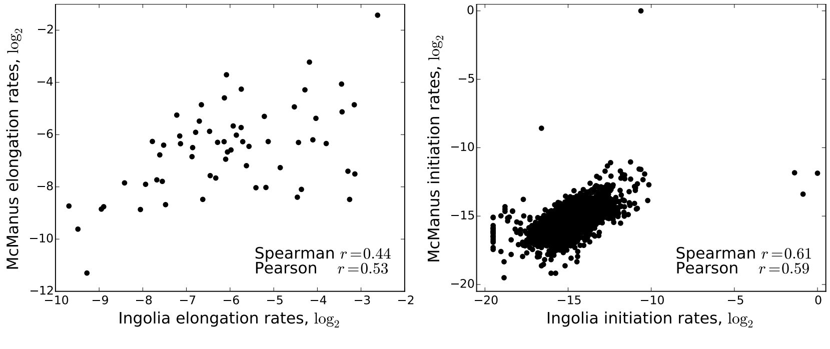

Figure SI-19. Translation elongation rates (left) and translation initiation rates (right) fitted independently on the McManus and Ingolia datasets also show qualitatively similar behaviour (Pearson $r = 0.531$, $p < 10^{-4}$ for elongation rates; and $r = 0.592$, $p < 10^{-277}$ for initiation rates).

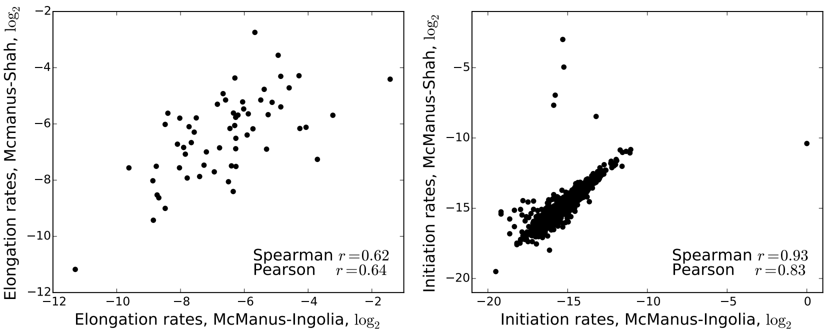

Figure SI-20. Translation elongation rates (left) and translation initiation rates (right) fitted independently on different sets of genes from McManus datasets show similar behaviour (Pearson $r = 0.644$, $p < 10^{-7}$ for elongation rates; and $r = 0.830$, $p < 10^{-293}$ for initiation rates). As expected, elongation rates fitted on different sets of genes from the McManus dataset agree well with each other.

SI-12.2 Model fitting is robust to changes in the genes used for fitting

We also sought to compare the McManus translation rates obtained in the previous section to the rates we determined on the set of genes common between the McManus dataset and the SMoPT model. Translation rates for these two sets of genes quantitatively agree with each other (~~Figure~~Fig SI-20). This suggests that the rate fitting procedure is robust to the gene set used to obtain the rates. However, the agreement between the rates is not perfect. This could be a result of overfitting the rates in individual CV folds due to using only a single fold for training - a limitation dictated by the computational complexity of the fitting procedure.

Alternatively the non-perfect agreement may also be a consequence of the implicit assumption, that codon elongation rates are independent of codon context, being incorrect. All translation models proposed to date, including ours, assume that translation elongation rates are constant and are not influenced by the sequence around a particular codon, although various factors affecting the speed of elongation have been suggested [17]. Differences between elongation rates induced by local sequences that are over-represented in a particular gene set provide an alternative explanation for the limited agreement between translation rates fitted on different sets.

SI-13 Model fitting without segment trees

In order to assess the effect of gene segmentation on the fitted translation rates we sought to compare our model to one fitted without the use of segment trees. To this end we restricted our segment trees to a maximal depth of 1 (i.e. they were allowed to contain only the top-level segment, corresponding to the entire CDS). The 4,768 full-CDS segments obtained in such way were bias-corrected as described in Section SI-4 (results are shown in ~~Figures~~Figs SI-21 and SI-22), and a single shape parameter $\sigma \approx 0.17$ ($\log_2$ scale) was estimated for all full-CDS segments. The set of full-CDS segments was then used to fit the TASEP^{init} model and compare its predictions to the independent PA datasets and to the TASEP^{init} model fitted with the use of segment trees.
It can be seen from ~~Figure~~Fig SI-23 that (a) predictions of the full-CDS model compare favourably to the predictions made by the original TASEP^{init} model; and that (b) the predicted ribosome occupancy of the full-CDS model and the measured per-transcript density are also in agreement. A cloud of outlier points that can be clearly seen in ~~Figure~~Fig SI-23 (right) consists of genes with low fitted initiation rates. This suggests that the initiation rate approximation procedure used for segment trees may not be very suitable for the case when only the full-CDS segments are used. Table SI-6 shows that the correlation

between the predictions made by the full-CDS TASEP^{init} model and independent PA datasets are lower, but comparable to the correlations obtain by that model fitted on segment trees. Together these findings suggest that the use of segment trees, when compared to the traditional full-CDS approach, does not introduce any significant biases into the fitted rates, but instead makes the initiation rate approximation procedure more accurate.

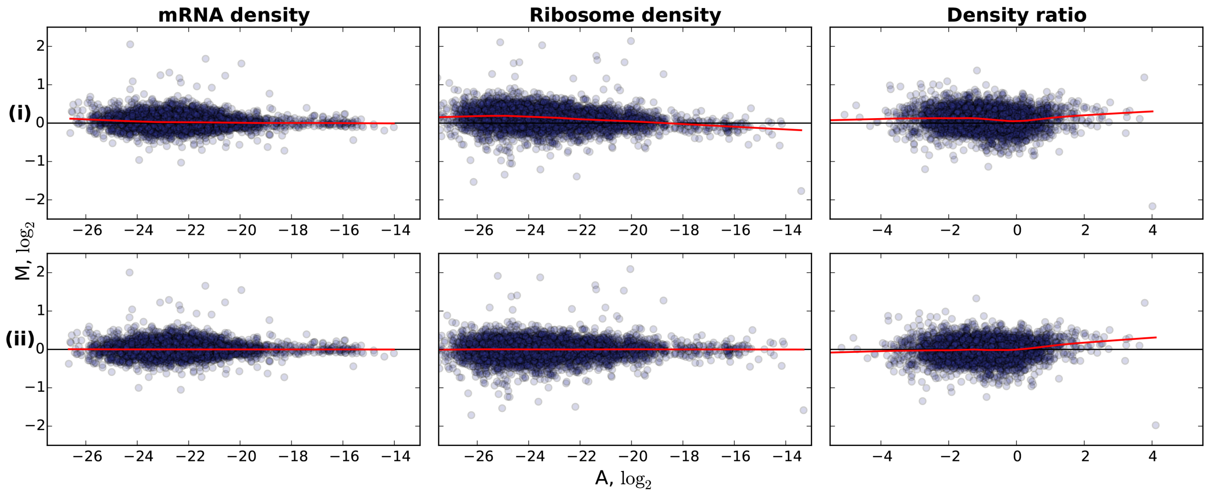

Figure SI-21. MA-plots of the full-CDS density and ratio measurements for the McManus *et al.* [1] dataset (i) before bias correction show a density-dependent systematic bias, which (ii) after removal of mRNA and ribosome density bias is no longer present in density estimates. An identical procedure is applied to correct the density-dependent bias amplified in ratio measurements (see ~~Figure~~Fig SI-17).

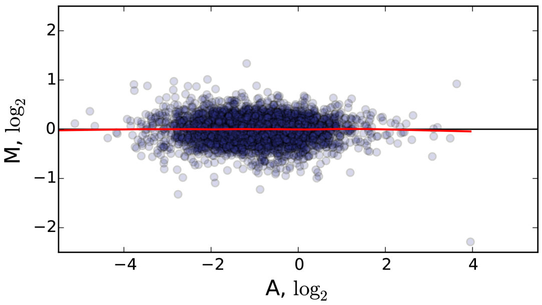

Figure SI-22. MA-plot of the full-CDS density ratio estimates of the McManus *et al.* [1] dataset after normalization shows that no significant bias is present.

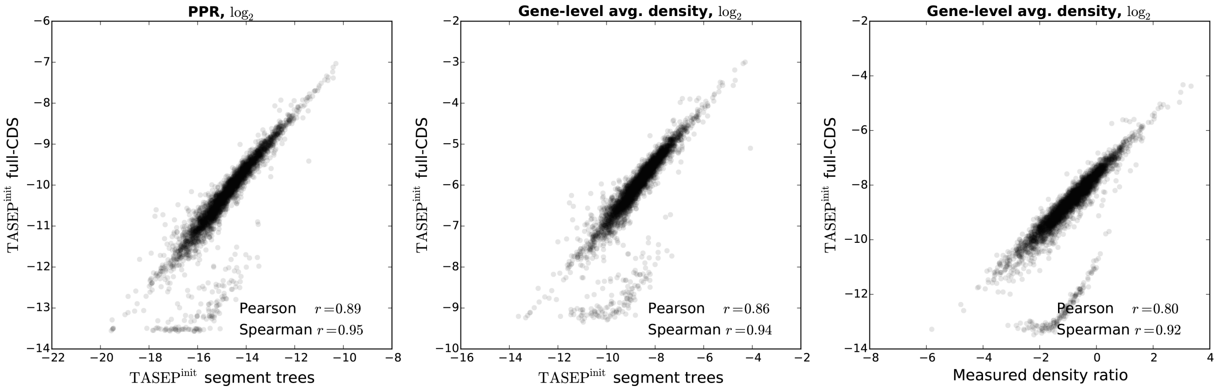

Figure SI-23. PPR (left) and gene-level average density (middle) predicted by the TASEP^{init} model fitted on full-CDS segments and the TASEP^{init} fitted on segment trees agree well with each other. Similarly, gene-level average density predicted by TASEP^{init} fitted on full-CDS estimates agrees well with density ratios obtained from RP data (right).

Table SI-6. Correlations of TASEP^{init} predictions with independent PA datasets for the full-CDS model. Spearman rank correlation coefficients r for are reported; J' is the partial correlation between J and PA given mRNA.

	TASEP^{init} full-CDS		
	Newman YEPD	Newman SD	Ghaemmaghami
Init. rate	$r = 0.56^{***}$	$r = 0.55^{***}$	$r = 0.48^{***}$
J	$r = 0.57^{***}$	$r = 0.55^{***}$	$r = 0.48^{***}$
$J \times$ mRNA	$r = 0.71^{***}$	$r = 0.69^{***}$	$r = 0.61^{***}$
J'	$r = 0.50^{***}$	$r = 0.47^{***}$	$r = 0.35^{***}$

* - $p-$value $< 10^{-5}$ ** - $p-$value $< 10^{-20}$ *** - $p-$value $< 10^{-100}$

We also used the set of full-CDS segments to fit elongation and initiation rates of the TASEP^{elong} model as described before. The best fits (note that the CMA evolutionary strategy will at best find one of the many equally good solutions if the problem is underdetermined) from each of the folds were used to calculate the CV mean rate and its SD for each of the 61 codons (shown in ~~Figure~~Fig SI-24). It can be seen from ~~Figure~~Fig SI-24 that fitted elongation rates differ considerably between CV folds, suggesting that the full-CDS approach does not provide sufficient constraints for simultaneously determining translation elongation and translation initiation rates of the full-CDS TASEP^{elong} model.

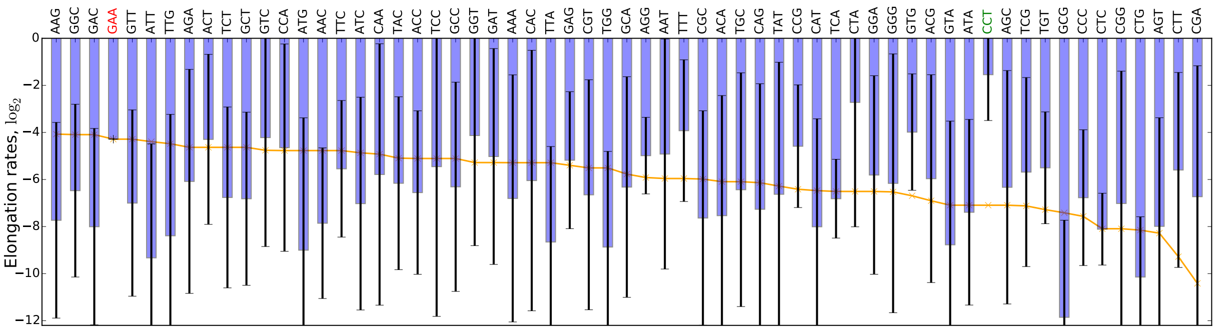

Figure SI-24. Mean and SD of the codon elongation rates fitted in different CV folds of the McManus dataset using the full-CDS approach, compared to the tAI-based rates (orange line). For most codons fitted elongation rates show large variation between CV folds.

SI-14 Functional enrichment

Gene ontology functional enrichment analysis was performed using the DAVID tool [18] with functional categories `GOTERM_BP_FAT`, `GOTERM_CC_FAT` and `GOTERM_MF_FAT`. A score cutoff of 0.1 and a size cutoff of 2 were used in the analysis. Only enrichments significant at 0.05 FDR were reported.

SI-15 Segment tree reconstruction

To visualize the change of density along transcripts and the uncertainty about it captured by the segment trees, we sought to obtain a reconstruction of the per-transcript ribosome density of the tree, which could be directly plotted. Since every node within the segment tree defines a probability distribution (PD) of the average density ratio of the corresponding segment, together these segments define a joint probability distribution of segment-averaged (i.e. piecewise constant) density of the entire gene. Samples from this joint PD can be used to reconstruct the encoded density and to obtain confidence bounds on the reconstruction. We note that the logarithm of the PDF of this distribution has the same form as the objective function ψ evaluated for the same gene.

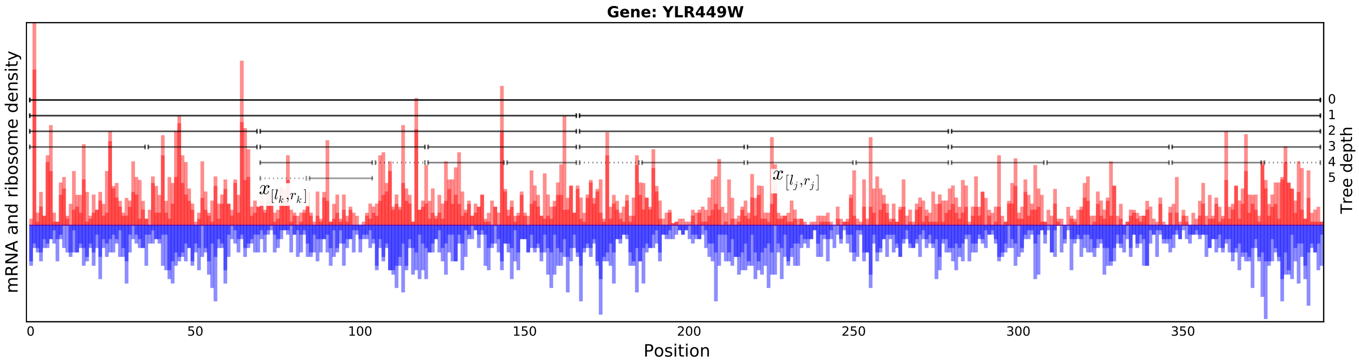

Figure SI-25. Segment tree shown for gene YLR449W visualized as in the main text. Segments from the segment tree (solid lines) are assigned variables $x_{[l_j,r_j]}$, whereas segments that need to be added to the tree (dotted lines) are assigned variables $x_{[l_k,r_k]}$.

Formally we assign a random variable $x_{[l_j,r_j]}$, describing the average density of the corresponding

segment to every leaf segment $[l_j, r_j]$ in the tree, and a random variable $x_{[l_k r_k]}$ with the same meaning to every segment $[l_k, r_k]$ that needs to be added to the tree in order for each parent node to have exactly two children (see ~~Figure~~Fig SI-25). These variables are used to compute the average density at every non-leaf segment as the weighted mean of values $x_{[l_j,r_j]}$ falling within that segment with segment lengths used as weights. We then assume a wide uniform prior for variables x and use Markov chain Monte Carlo [19] to sample them from the joint PD.

When building reconstructions for visualization we obtained 2×10^8 samples with a burn phase of 10^8 samples and thinning of 100, yielding a total of 10^6 samples. The 10%, 50% and 90% highest posterior density (HPD) intervals calculated from this sample were then used to plot the reconstruction.

References

1. McManus CJ, May GE, Spealman P, Shteyman A (2014) Ribosome profiling reveals post-transcriptional buffering of divergent gene expression in yeast. Genome research 24: 422–430.

2. Ingolia NT, Lareau LF, Weissman JS (2011) Ribosome profiling of mouse embryonic stem cells reveals the complexity and dynamics of mammalian proteomes. Cell 147: 789–802.

3. Langmead B, Trapnell C, Pop M, Salzberg SL, et al. (2009) Ultrafast and memory-efficient alignment of short DNA sequences to the human genome. Genome Biol 10: R25.

4. Cherry JM, Hong EL, Amundsen C, Balakrishnan R, Binkley G, et al. (2011) Saccharomyces Genome Database: the genomics resource of budding yeast. Nucleic acids research : gkr1029.

5. Yang YH, Dudoit S, Luu P, Lin DM, Peng V, et al. (2002) Normalization for cDNA microarray data: a robust composite method addressing single and multiple slide systematic variation. Nucleic acids research 30: e15–e15.

6. Ingolia NT, Ghaemmaghami SA, Newman JRS, Weissman JS (2009) Genome-Wide Analysis in Vivo of Translation with Nucleotide Resolution Using Ribosome Profiling. Science 324: 218–223.

7. Cleveland WS (1979) Robust locally weighted regression and smoothing scatterplots. Journal of the American statistical association 74: 829–836.

8. Siwiak M, Zielenkiewicz P (2010) A comprehensive, quantitative, and genome-wide model of translation. PLoS computational biology 6: e1000865.

9. Ciandrini L, Stansfield I, Romano MC (2013) Ribosome traffic on mRNAs maps to gene ontology: genome-wide quantification of translation initiation rates and polysome size regulation. PLoS computational biology 9: e1002866.

10. Shah P, Ding Y, Niemczyk M, Kudla G, Plotkin JB (2013) Rate-Limiting Steps in Yeast Protein Translation. Cell 153: 1589–1601.

11. Yates RD, Goodman DJ (2005) Probability and Stochastic Processes: A Friendly Introduction for Electrical and Computer Engineers. John Wiley & Sons, 2nd edition.

12. Zenklusen D, Larson DR, Singer RH (2008) Single-RNA counting reveals alternative modes of gene expression in yeast. Nature structural & molecular biology 15: 1263–1271.

13. Miura F, Kawaguchi N, Yoshida M, Uematsu C, Kito K, et al. (2008) Absolute quantification of the budding yeast transcriptome by means of competitive PCR between genomic and complementary DNAs. BMC genomics 9: 574.

14. Zhang G, Ignatova Z (2009) Generic algorithm to predict the speed of translational elongation: implications for protein biogenesis. PLoS One 4: e5036.

15. Wohlgemuth SE, Gorochowski TE, Roubos JA (2013) Translational sensitivity of the *Escherichia coli* genome to fluctuating tRNA availability. Nucleic acids research 41: 8021–8033.

16. Raabe CA, Tang TH, Brosius J, Rozhdestvensky TS (2014) Biases in small RNA deep sequencing data. Nucleic acids research 42: 1414–1426.

17. Tuller T, Veksler-Lublinsky I, Gazit N, Kupiec M, Ruppin E, et al. (2011) Composite effects of gene determinants on the translation speed and density of ribosomes. Genome Biol 12: R110.

18. Huang DW, Sherman BT, Lempicki RA (2009) Bioinformatics enrichment tools: paths toward the comprehensive functional analysis of large gene lists. Nucleic acids research 37: 1–13.

19. Patil A, Huard D, Fonnesbeck CJ (2010) PyMC: Bayesian stochastic modelling in Python. Journal of statistical software 35: 1.
