# Supplementary figures and images for: Unbiased Quantitative Models of Protein Translation Derived from Ribosome Profiling Data
Source: PLoS Comput Biol. 2015 Aug 14;11(8):e1004336. doi: 10.1371/journal.pcbi.1004336 (PMC4537299; doi:10.1371/journal.pcbi.1004336)

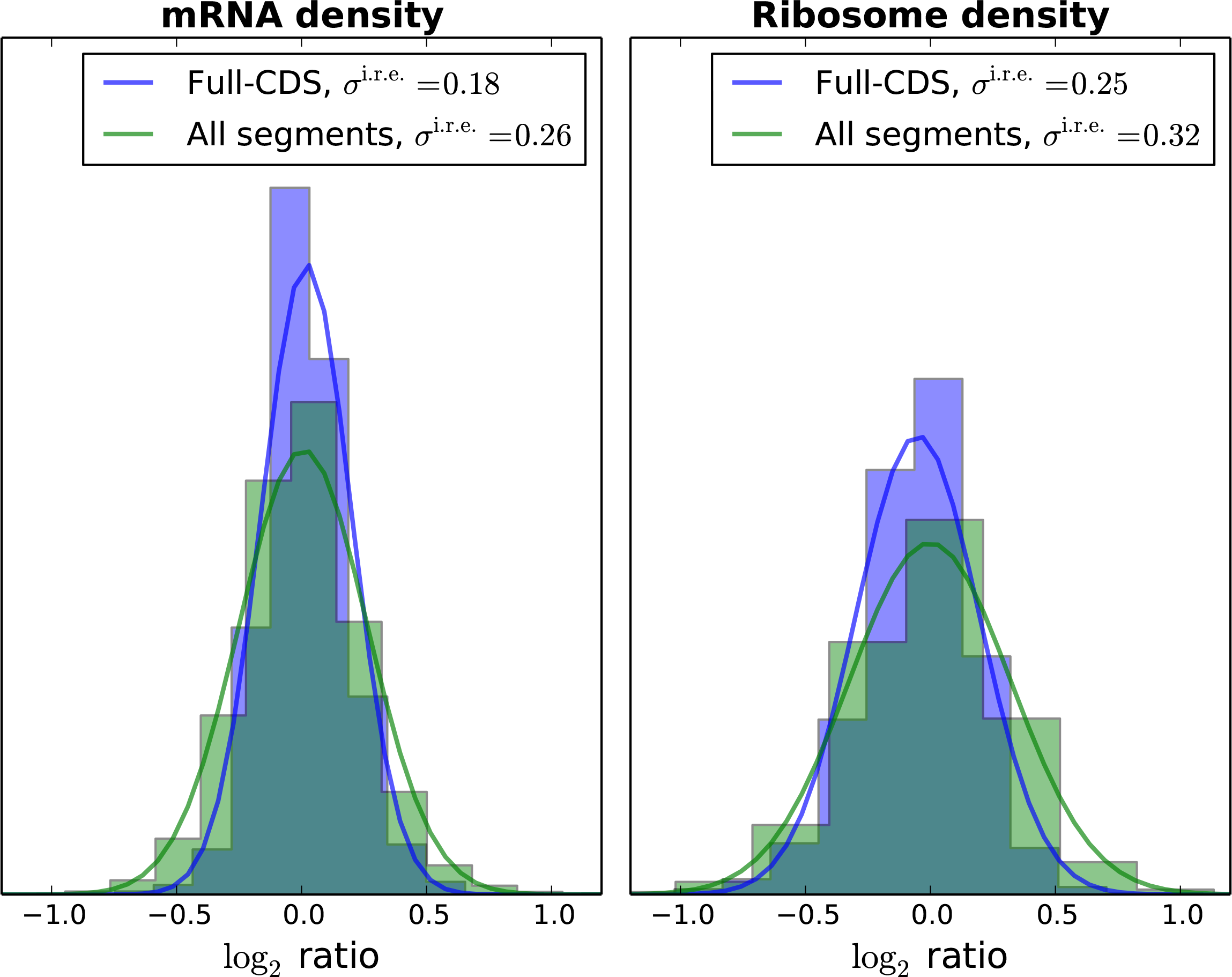

Supplement: S1 Fig — Distributions fitted into data (solid lines) are centered around zero, but their SDs differ. (TIF) [file pcbi.1004336.s002.tif]

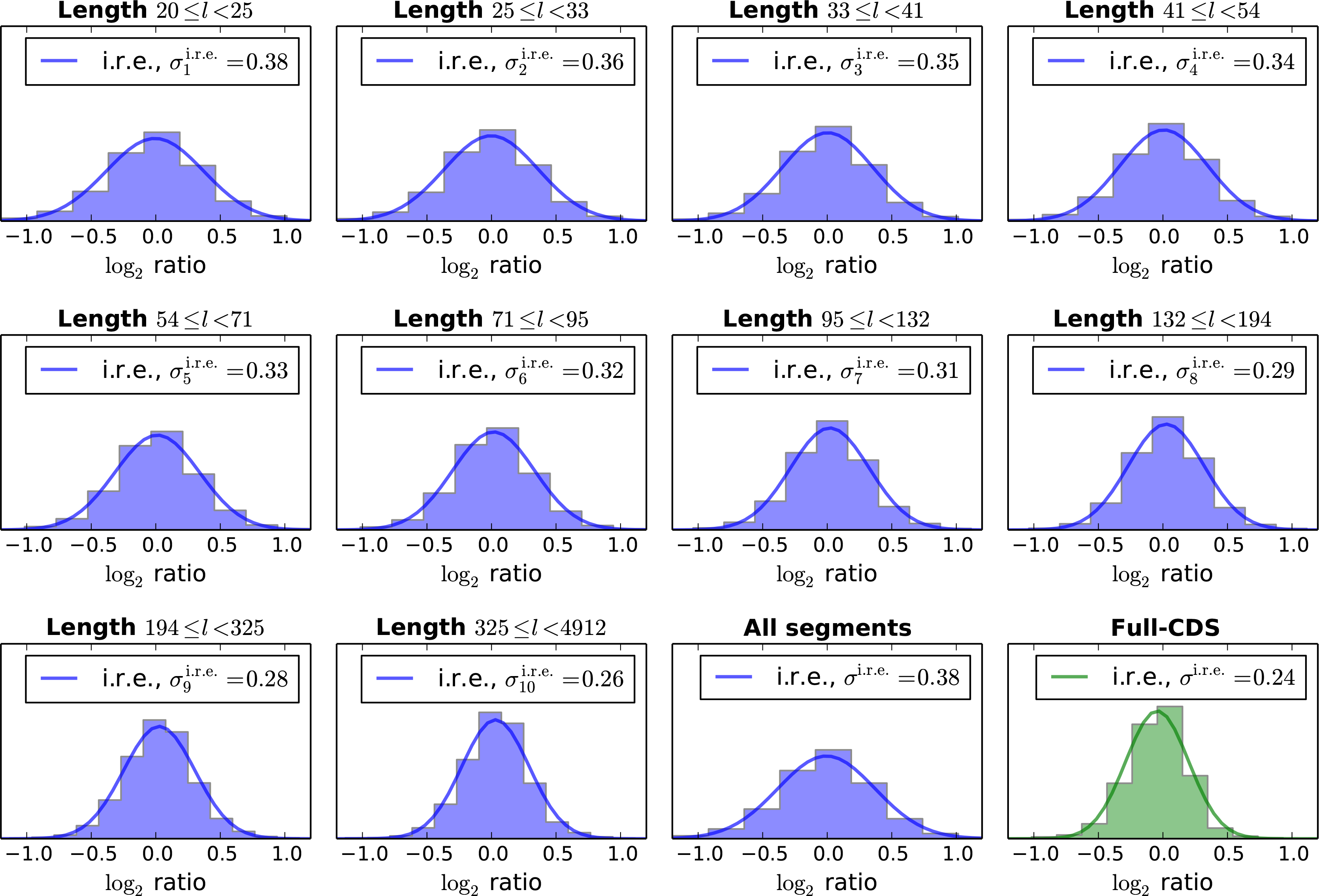

Supplement: S2 Fig — The group shape parameters of the i.r.e. and the density ratio distributions are related as σkgroup=12σki.r.e.. (TIF) [file pcbi.1004336.s003.tif]

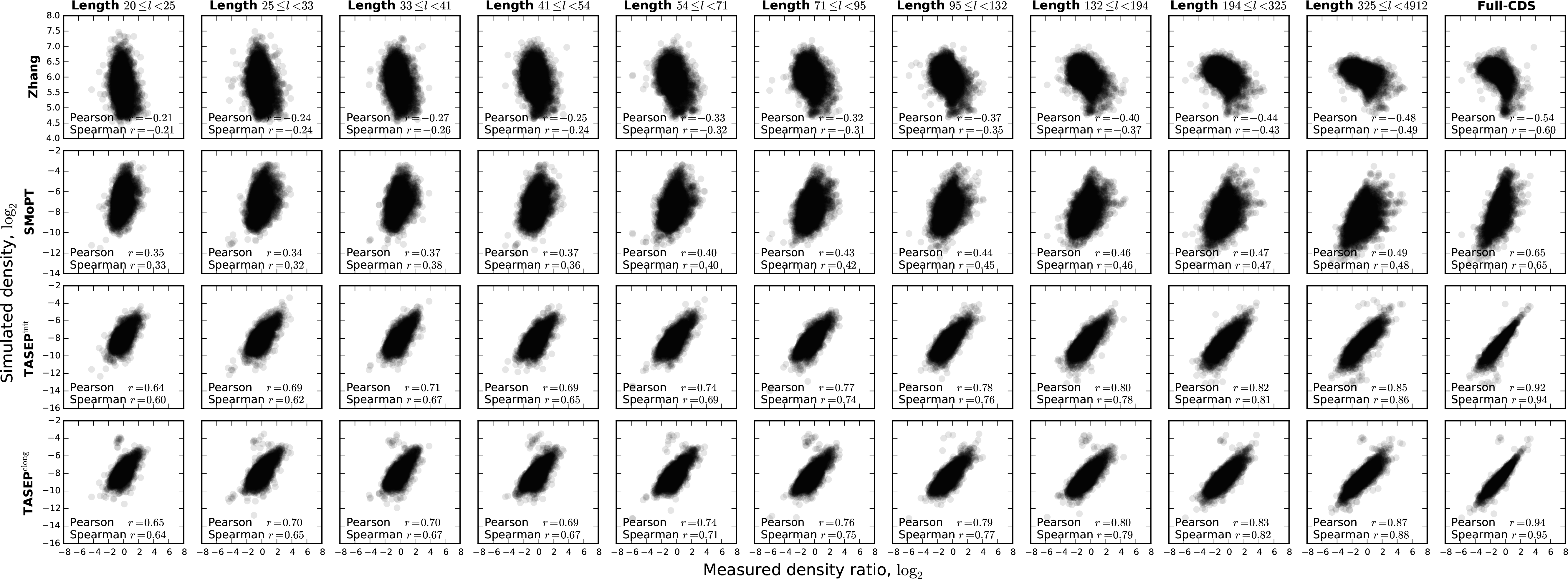

Supplement: S3 Fig — TASEPinit and TASEPelong significantly improve over existing models for all segment length groups. (TIF) [file pcbi.1004336.s004.tif]

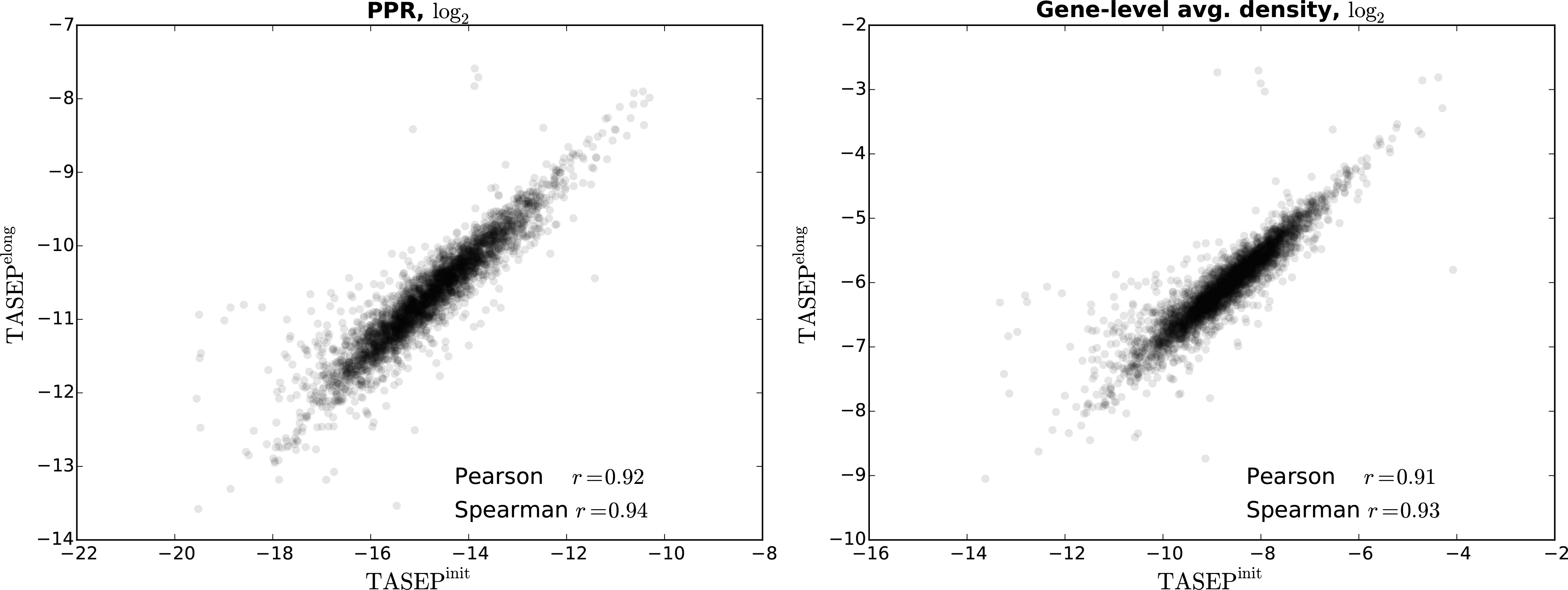

Supplement: S4 Fig — (TIF) [file pcbi.1004336.s005.tif]

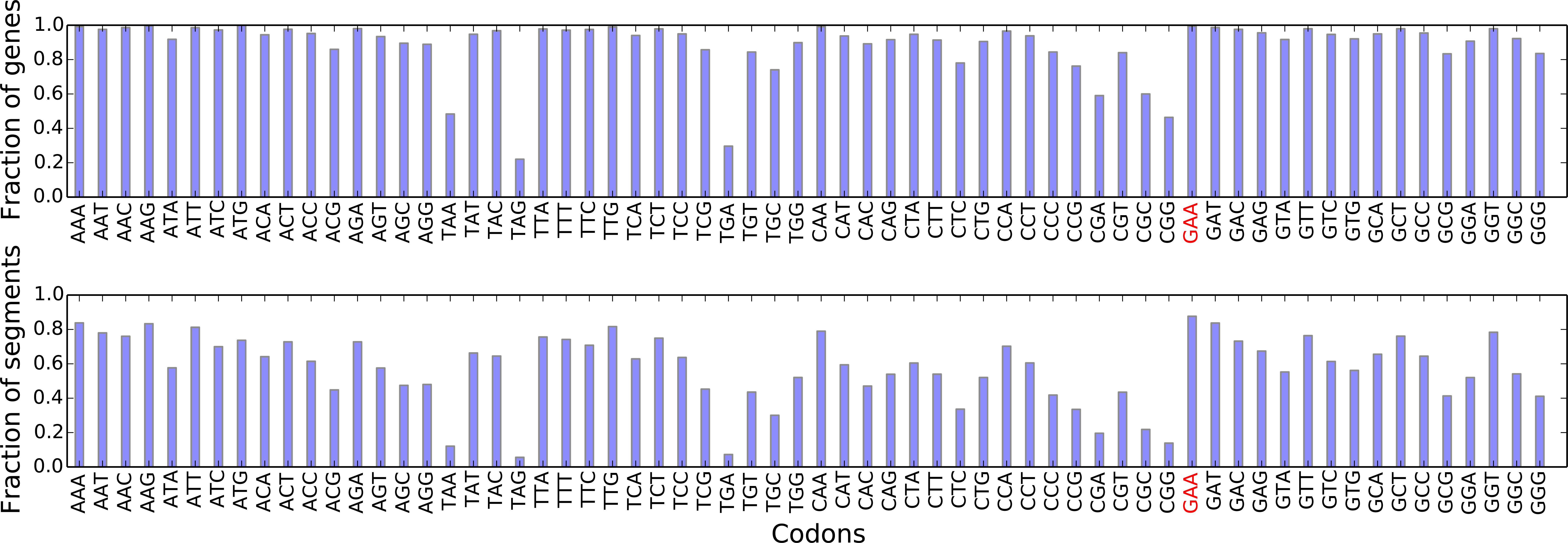

Supplement: S5 Fig — Translation rate of codon GAA (red) was fixed in elongation rate fitting experiments as it is present in many genes and segments. (TIF) [file pcbi.1004336.s006.tif]

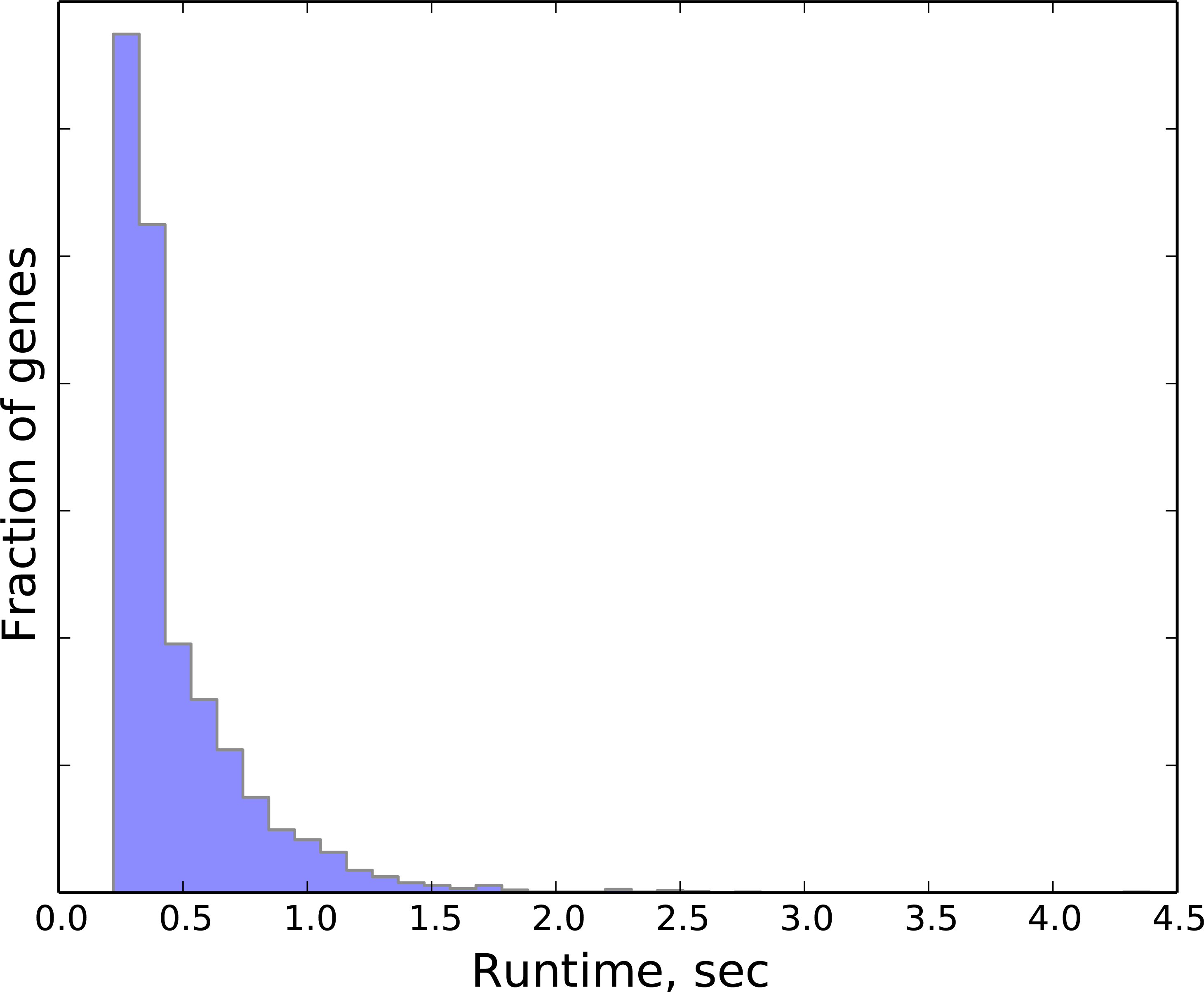

Supplement: S6 Fig — tAI-based elongation rates and initiation rates of 1.0 were used in the simulations. (TIF) [file pcbi.1004336.s007.tif]
