# Supplementary material for: Unbiased Quantitative Models of Protein Translation Derived from Ribosome Profiling Data
Source: PLoS Comput Biol. 2015 Aug 14;11(8):e1004336. doi: 10.1371/journal.pcbi.1004336 (PMC4537299; doi:10.1371/journal.pcbi.1004336)
Supplement: S1 Table — Left (inclusive) and right (exclusive) edges give the range of segment lengths of a given group. (PDF) [file pcbi.1004336.s008.pdf]

Table S1: Shape parameters of the density ratio distributions for segments grouped by length. Left (inclusive) and right (exclusive) edges give the range of segment lengths of a given group.

#	Left	Right	Group size	Shape parameter σ, $\log_2$
1	20	25	5284	0.235565455789103
2	25	33	6804	0.216285904079930
3	33	41	5591	0.207713921552678
4	41	54	6361	0.199316542112745
5	54	71	6163	0.183918710525198
6	71	95	5989	0.177805660887211
7	95	132	6097	0.164353210454788
8	132	194	6063	0.159682537763375
9	194	325	6057	0.142796300946171
10	325	4912	6057	0.128137654321086
